# Prediction of outpatient rehabilitation patient preferences and optimization of graded diagnosis and treatment based on XGBoost machine learning algorithm

**DOI:** 10.3389/frai.2024.1473837

**Published:** 2025-01-15

**Authors:** Xuehui Fan, Ruixue Ye, Yan Gao, Kaiwen Xue, Zeyu Zhang, Jing Xu, Jingpu Zhao, Jun Feng, Yulong Wang

**Affiliations:** ^1^Department of Rehabilitation Medicine, The First Affiliated Hospital of Shenzhen University, The Second People’s Hospital of Shenzhen, Shenzhen, Guangdong, China; ^2^Linping Hospital of Integrated Traditional Chinese and Western, Medicine, Hangzhou, Zhejiang, China

**Keywords:** XGBoost, machine learning algorithm, rehabilitation patient, graded diagnosis and treatment, treatment preferences

## Abstract

**Background:**

The Department of Rehabilitation Medicine is key to improving patients’ quality of life. Driven by chronic diseases and an aging population, there is a need to enhance the efficiency and resource allocation of outpatient facilities. This study aims to analyze the treatment preferences of outpatient rehabilitation patients by using data and a grading tool to establish predictive models. The goal is to improve patient visit efficiency and optimize resource allocation through these predictive models.

**Methods:**

Data were collected from 38 Chinese institutions, including 4,244 patients visiting outpatient rehabilitation clinics. Data processing was conducted using Python software. The pandas library was used for data cleaning and preprocessing, involving 68 categorical and 12 continuous variables. The steps included handling missing values, data normalization, and encoding conversion. The data were divided into 80% training and 20% test sets using the Scikit-learn library to ensure model independence and prevent overfitting. Performance comparisons among XGBoost, random forest, and logistic regression were conducted using metrics, including accuracy and receiver operating characteristic (ROC) curves. The imbalanced learning library’s SMOTE technique was used to address the sample imbalance during model training. The model was optimized using a confusion matrix and feature importance analysis, and partial dependence plots (PDP) were used to analyze the key influencing factors.

**Results:**

XGBoost achieved the highest overall accuracy of 80.21% with high precision and recall in Category 1. random forest showed a similar overall accuracy. Logistic Regression had a significantly lower accuracy, indicating difficulties with nonlinear data. The key influencing factors identified include distance to medical institutions, arrival time, length of hospital stay, and specific diseases, such as cardiovascular, pulmonary, oncological, and orthopedic conditions. The tiered diagnosis and treatment tool effectively helped doctors assess patients’ conditions and recommend suitable medical institutions based on rehabilitation grading.

**Conclusion:**

This study confirmed that ensemble learning methods, particularly XGBoost, outperform single models in classification tasks involving complex datasets. Addressing class imbalance and enhancing feature engineering can further improve model performance. Understanding patient preferences and the factors influencing medical institution selection can guide healthcare policies to optimize resource allocation, improve service quality, and enhance patient satisfaction. Tiered diagnosis and treatment tools play a crucial role in helping doctors evaluate patient conditions and make informed recommendations for appropriate medical care.

## Background

In the contemporary healthcare system, the choice of medical services by patients has increasingly become an important area of research, which not only relates to individual health outcomes but also affects the resource allocation and efficiency of the entire healthcare system. With economic development, an aging population, and the diversification of healthcare service demands, patients’ preferences when selecting medical services have shown increasingly complex characteristics. Patients desire more personalized medical services to meet their specific health needs (Chatwiriyaphong et al., 2024; Guo et al., 2024). This shift has prompted researchers to focus on various factors influencing patient choices, including socioeconomic status, education level, past medical experiences, and perceptions of healthcare services.

Existing literature has demonstrated that different demographic characteristics and psychological backgrounds significantly impact patients’ medical choices. For instance, studies have shown that patients with better economic conditions tend to choose higher-level medical institutions, while low-income patients may prefer low-cost services (Bie et al., 2024). Additionally, patients’ education levels have been found to correlate with their choice of medical institutions; those with higher education levels are generally more inclined to select reputable medical institutions and seek more specialized medical services (Zhang et al., 2015). Although previous research has revealed some factors influencing patients’ medical choices, studies on patient choice behavior in specific treatment processes (such as outpatient rehabilitation) remain relatively limited (Wang et al., 2018). Particularly in the context of China, research involving outpatient rehabilitation patients is scarce, highlighting the urgent need to fill this knowledge gap.

The core objective of this study is to explore the main factors influencing Chinese patients’ choices of medical institutions in surgical rehabilitation outpatient settings and how these factors interact. This study will focus on analyzing key variables affecting patient decision-making, such as age, gender, economic status, educational background, insurance coverage, and past medical experiences. Furthermore, we will investigate patients’ overall satisfaction with medical services and its impact on their healthcare choices. A comprehensive analysis of these factors will not only help clarify the decision-making processes of patients but also provide guidance for healthcare institutions in resource allocation and service optimization.

This study will employ a large-scale survey method, covering 4,244 patients from 38 medical institutions. A predictive model established using the XGBoost algorithm will be utilized to analyze the complex factors influencing patient choices in depth. By analyzing this data, we aim to identify potential decision-making patterns, providing crucial insights for healthcare providers to understand patient needs. Additionally, we will develop a rehabilitation grading tool based on the Daily Life Ability Scale (Longshi Scale) to accurately quantify patients’ rehabilitation needs and the severity of their conditions. We have included the triage assessment tool in Supplementary material. This grading tool has been embedded in our program when used. When used by doctors, the triage assessment results are automatically generated. This tool will assist doctors in making more precise decisions when assessing and recommending medical institutions, enhancing patients’ healthcare experiences, and optimizing the allocation of medical resources. By integrating modern data analysis techniques with advancements in clinical medicine, our research will not only improve the understanding of patient choice behavior but also promote the improvement of outpatient rehabilitation processes, enabling patients to receive more suitable medical services (Wang et al., 2019).

In summary, although there is a certain foundation of research on patient medical choices, systematic analysis in the field of surgical rehabilitation remains insufficient. This study aims to provide new perspectives and insights in this area through rigorous scientific methods, with the expectation of making substantial contributions to enhancing patients’ healthcare experiences and health outcomes.

## Methods

### Participant selection and ethical review

This study followed a strict ethical review process to ensure that the research design complied with international ethical standards. Prior to the commencement of the study, we obtained approval from the institutional ethics review board. All participants were required to sign an informed consent form before joining the study to ensure their understanding of the research purpose, procedures, potential risks, and rights, including the right to withdraw from the study.

Inclusion criteria: Participants must have clear rehabilitation requirements and detailed outpatient medical records. Additionally, all participating physicians must hold valid licenses and possess a minimum of 3 years of relevant clinical experience.

Exclusion criteria: Individuals who were unable to complete the assessment tasks required for the study, including those who could not regularly attend follow-up visits because of health issues or other personal reasons, were excluded.

Withdrawal criteria: This included cases in which participants experienced severe adverse events, compliance issues, or chose to voluntarily withdraw from the study.

All variables involved in the study are listed in Supplementary materials 2–4.

The tiered diagnostic assessment scale we designed divided patients in rehabilitation clinics into six assessment levels: Assessment scores range from 3 to 9 points. Level 1–2 defines Bedridden group. A score below 4 indicates a complete inability to manage daily life, while a score of 4–9 indicates a basic inability to manage daily life; Level 3–4 defines Domestic group. A score below 4 indicates partial self-sufficiency, while a score of 4–9 indicates greater self-sufficiency; Level 5–6 defines Community group. A score below 4 indicates basic self-sufficiency, while a score of 4–9 indicates complete self-sufficiency. Detailed explanations of each level and the corresponding scores are provided in the Supplementary material 5.

### Data collection and preprocessing

Python was used for data processing.^1^ Initially, we extracted data from structured electronic medical records and imported them into an Excel spreadsheet. We established 68 categorical variables and 12 continuous variables; the specific variables are listed in Supplementary material. Subsequently, we loaded the data using the pandas library in Python and conducted data cleaning and preprocessing (see text footnote 1). During the data cleaning process, non-essential variables were removed and all missing values were handled. For numerical variables, we employed mean imputation to address missing values; whereas for categorical variables, specific values were filled according to actual circumstances or new categories were created to represent “data missing.” Finally, we used the Scikit-learn library^2^ to split the data into an 80% training set and 20% test set to ensure independence for model training and evaluation while preventing overfitting.

### Model selection and performance comparison

We conducted a preliminary comparison of three mainstream machine learning models: XGBoost, random forest, and logistic regression. The training and evaluation processes for these models were implemented using the Scikit-learn library. The performance evaluation of the models was based on multiple metrics, such as accuracy and receiver operating characteristic (ROC) curves. To address the issue of sample imbalance, we utilized the imbalanced-learn library^3^ to complete the training process for each model, using a pipeline that included data preprocessing and the SMOTE technique.

### Parameter settings for machine learning models and SMOTE technique

In this study, we compared three machine learning models: XGBoost, Random Forest, and Logistic Regression. Ultimately, the XGBoost model, which demonstrated the best performance, was selected. The parameter settings for the XGBoost model are as follows:

**XGBoost Model:**

- max_depth: 5
- learning_rate: 0.1
- n_estimators: 200
- scale_pos_weight: 10
- eval_metric: “mlogloss”
- random_state: 42

The parameter settings for the Random Forest model are as follows:

**Random Forest:**

- n_estimators: 200
- max_depth: 5
- random_state: 42

The Logistic Regression model was implemented with default parameters.

To address the issue of class imbalance, we applied the SMOTE (Synthetic Minority Oversampling Technique) during the model training process. The specific parameters for SMOTE are as follows:

- sampling_strategy: “auto”
- random_state: 42

SMOTE was integrated into the preprocessing phase of the training data to mitigate class imbalance by generating synthetic samples.

### Model analysis and optimization

After selecting the XGBoost model with the best performance, a further in-depth analysis was conducted. By evaluating the model’s confusion matrix, we not only confirmed its classification effectiveness but also identified the most influential factors on the prediction results by calculating the importance of each feature. Partial dependence plots (PDP) were used to reveal the relationship between the key predictive variables and the target variable.

### Results interpretation and analysis

The study results are visually presented in various forms, such as bar charts, ROC curves, confusion matrices, and PDP, to highlight the decision logic and performance of the XGBoost model.

## Results

### Model performance overview

The aim of this study was to evaluate the performance of four different machine learning models in classification tasks: XGBoost, random forest, and logistic regression. The dataset contains 849 samples spanning nine categories. During the evaluation process, the XGBoost model demonstrated a high overall accuracy rate of 80.21%. This model performed best in Category 1, with a precision of 83% and a recall of 91%. However, its performance was poor in Category 6, with a recall rate of only 9% and a precision rate of 33%. This suggests that the XGBoost model exhibits some imbalance when dealing with categories of different scales, particularly when performing lower for smaller-scale categories. Similarly, the overall accuracy rate for the random forest model closely approximated that of XGBoost at 80.33%.

The model performed similarly to XGBoost in most categories, achieving a 92% recall rate for Category 1. However, the random forest model failed to make any valid predictions in Category 8, with precision and recall rates of 0. The performance of the logistic regression model was significantly lower than that of the first two models, with an overall accuracy of only 21.67%. The model did not demonstrate effective classification abilities in any category, particularly in Category 1, where the recall rate was only 11%, despite the large number of samples. In the model comparison, the ensemble learning methods (XGBoost and random forest) outperformed the single models (logistic regression) on the dataset. This may be because of the ability of ensemble methods to generalize various features and data of different scale classes (Table 1).

**Table 1:** Comparison of performance metrics (accuracy, precision, recall, and F1-score) for three machine learning models: XGBoost, random forest, and logistic regression.

Comparison of XGBoost, random forest, and logistic regression					
Model	Accuracy	Precision	Recall	F1-score	Support
XGBoost	0.802120141	0.83	0.91	0.87	431
XGBoost		0.79	0.58	0.67	77
XGBoost		0.8	0.84	0.82	102
XGBoost		0.74	0.75	0.75	154
XGBoost		0.84	0.64	0.73	42
XGBoost		0.33	0.09	0.14	11
XGBoost		0.64	0.41	0.5	22
XGBoost		1	1	1	1
XGBoost		0.83	0.56	0.67	9
Random forest	0.803297998	0.82	0.92	0.87	431
Random forest		0.77	0.56	0.65	77
Random forest		0.77	0.83	0.8	102
Random forest		0.79	0.76	0.77	154
Random forest		0.92	0.55	0.69	42
Random forest		1	0.09	0.17	11
Random forest		0.67	0.45	0.54	22
Random forest		0	0	0	1
Random forest		1	0.56	0.71	9
Logistic regression	0.216725559	0.59	0.11	0.18	431
Logistic regression		0.07	0.03	0.04	77
Logistic regression		0.23	0.62	0.34	102
Logistic regression		0.46	0.32	0.38	154
Logistic regression		0.12	0.21	0.16	42
Logistic regression		0.02	0.09	0.04	11
Logistic regression		0.11	0.32	0.16	22
Logistic regression		0	0	0	1
Logistic regression		0.03	0.56	0.06	9

**Figure 1:**
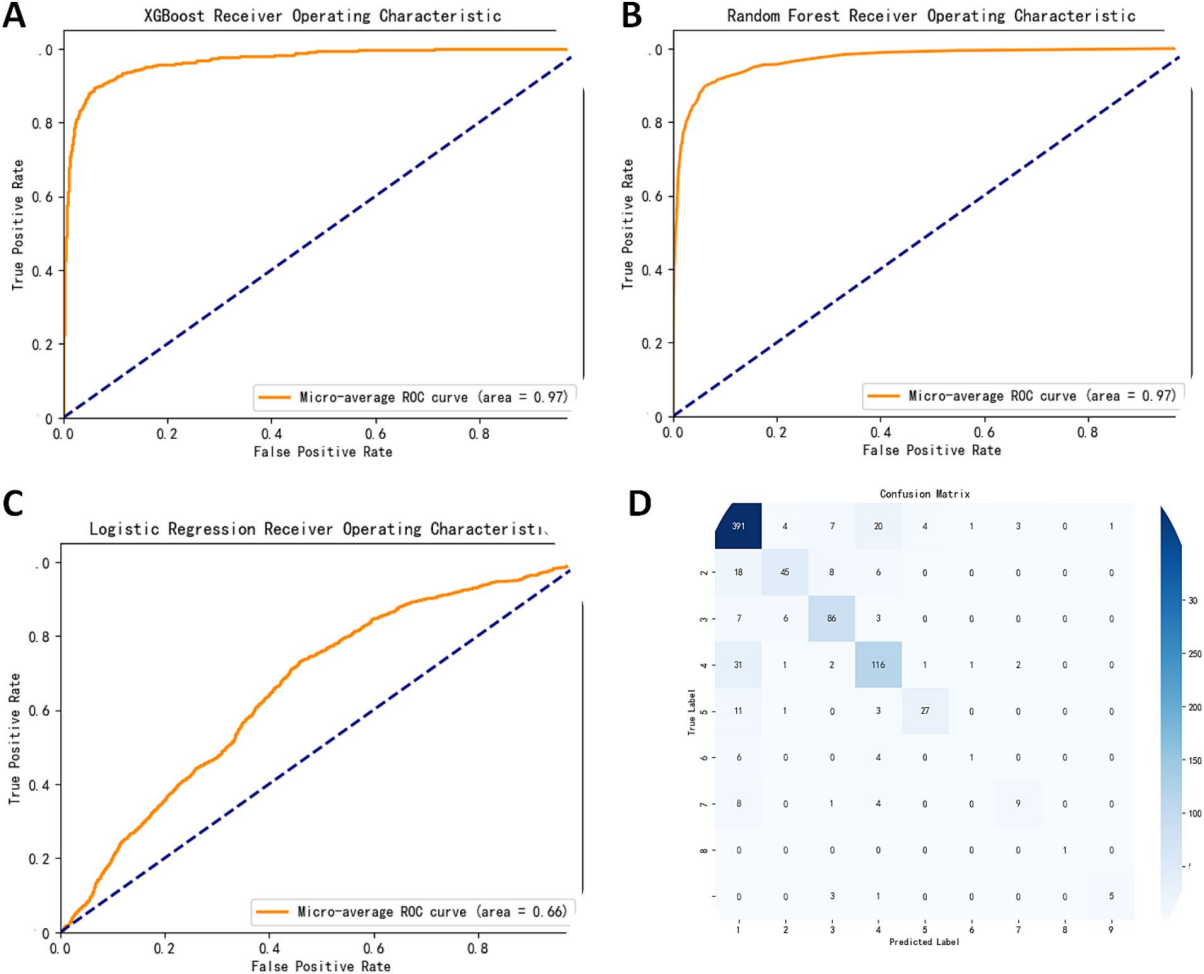
Performance evaluation of machine learning models. **(A)** ROC curve of the XGBoost model, with an AUC of 0.97, indicating excellent classification performance. **(B)** ROC curve of the Random Forest model, also with an AUC of 0.97, demonstrating similarly high classification capability. **(C)** ROC curve of the Logistic Regression model, with an AUC of 0.66, showing significantly lower classification performance compared to the other two models. The ROC curves depict the performance of three models [**(A)** XGBoost, **(B)** Random Forest, **(C)** Logistic Regression] in terms of their ability to distinguish between classes. The horizontal axis represents the False Positive Rate (FPR), and the vertical axis represents the True Positive Rate (TPR). The area under the curve (AUC) is provided in the legend, where higher AUC values indicate better classification performance. The diagonal dashed line represents random guessing (AUC = 0.5). The ROC curves demonstrate that both XGBoost **(A)** and Random Forest **(B)** achieve high AUC values of 0.97, while Logistic Regression **(C)** shows comparatively lower performance with an AUC of 0.66. **(D)** Confusion matrix illustrating the specific performance of the XGBoost model in classification tasks, with rows representing true labels and columns representing predicted labels. Darker cells indicate higher prediction accuracy.

Furthermore, ROC curves were plotted to evaluate the performance of the models. The results showed how different models performed in terms of the area under curve (AUC). The AUC value for the XGBoost model was 0.9684, indicating a good balance between the true-positive rate, false-positive rate, and excellent classification ability. The random forest model achieved an AUC value of 0.9693, demonstrating high performance. However, the logistic regression model’s AUC value is 0.6573, suggesting poor balance between true-positive and false-positive rates with relatively lower classification performance; potentially indicating underfitting or insufficient data features issues (Figures 1A–C). After comprehensive evaluation, we chose XGBoost as our best-performing model for this study (Supplementary material 7).

To further validate our findings, we employed K-fold stratified cross-validation to assess the performance of the models on handling imbalanced classification problems. This study utilized K-fold stratified cross-validation to evaluate the performance of the machine learning model in handling imbalanced classification problems. The cross-validation was conducted with five folds, and the results of each fold revealed fluctuations in the model’s performance across different evaluation metrics. The model’s average accuracy was 0.7877, indicating strong overall predictive capability. Meanwhile, the macro-averaged F1 score was 0.6284, highlighting the challenges the model faced in handling minority class samples. The F1 scores varied across the folds, with the fourth fold showing significantly better predictions for the minority class compared to the others, reflecting the model’s adaptability under certain conditions (Supplementary material 6, Image 1).

### Demonstration of confusion matrix and classification report based on the XGBoost model

In this study, we investigated a multiclass classification problem using the XGBoost model, and evaluated its performance using a confusion matrix. The results indicated that the XGBoost model demonstrated significant advantages in handling complex multiclass classification tasks. Specifically, we observed outstanding classification accuracy in Categories 1 and 4, successfully identifying 391 and 116 samples, respectively. This illustrates the strong identification capability of the model for the important categories. Despite some degree of misclassification in other categories, the model was still able to correctly identify a considerable number of samples, showing good generalization ability and robustness (Figure 1D). These findings strongly support the practical application of the XGBoost model and offer important references and methods for solving complex multiclass classification problems.

### Analysis of prediction results based on the XGBoost model: feature importance analysis

**Figure 2:**
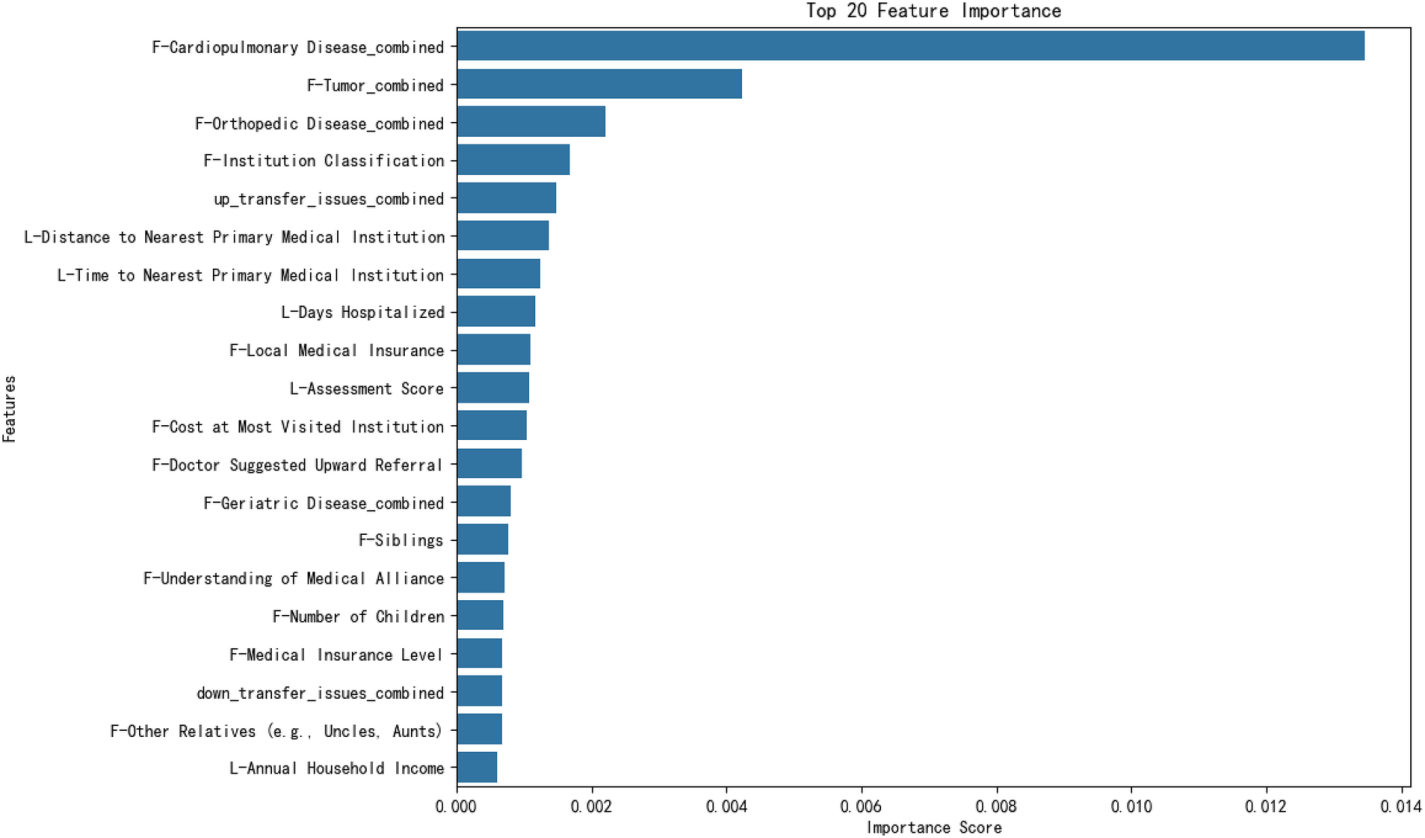
Feature importance ranking. The figure shows the importance scores of the top 20 features, ranked according to their impact on the prediction results. The horizontal axis represents the feature importance scores, and the vertical axis lists the features. This bar chart illustrates the top 20 features ranked by their importance scores in the machine learning model. The horizontal axis represents the importance score, which quantifies each feature’s contribution to the model’s predictions. The vertical axis lists the names of the features, with their font size increased for readability. Importance scores are computed based on the model’s evaluation metric, such as the feature split gain or frequency. The chart shows that the most influential features include “F-Cardiopulmonary Disease_combined,” “F-Tumor_combined,” and “F-Orthopedic Disease_combined,” which highlight health conditions as key predictors. Other features, such as distance and time to medical institutions, also contributed significantly to the predictions. Numerical annotations on the bars provide additional clarity regarding each feature’s relative importance.

Through an in-depth analysis of the importance of the characteristics of medical institution-level selection for rehabilitation outpatients, we found that patient selection behavior is influenced by multiple factors (Figure 2). First, the comprehensive feature ranking of cardiovascular and pulmonary diseases comes first, indicating that whether patients have heart- or lung-related diseases is crucial to their choice of medical institution. This may be because the serious impact of cardiovascular and pulmonary diseases on patients’ health conditions leads them to pay more attention and choose medical institutions that can provide related professional services. Second, the comprehensive features of tumors and orthopedic diseases rank second and third, respectively, indicating that whether patients have tumors or orthopedic diseases also has a significant impact on their choice of medical institution. This may reflect the serious impact of these diseases on the patients’ physical health and life, making them pay more attention to their choice of professional treatments and services. In addition, institutional grading ranked fourth in feature importance, showing that patients’ awareness of medical service quality and professional level is important for their selection behavior. The grading system may affect the patients’ trust in and preferences for medical institutions, thereby affecting their final choices.

After analyzing the importance of the features, we found that the selection of the medical institution level for patients choosing outpatient rehabilitation medicine was influenced by multiple factors. Assessment scores also play an important role in the selection process, in addition to individual patient health and medical service quality. These assessment scores are the result of evaluations conducted by doctors using the rehabilitation grading tool, which aims to quantify patient rehabilitation needs and severity of conditions to help doctors guide patients to appropriate medical institutions. Therefore, although the evaluation score, as part of the professional evaluation of doctors, ranks 10th in the importance of characteristics, it still has a significant impact on patients’ choice of medical institutions. This finding provides healthcare decision makers with comprehensive information that can assist in developing more effective healthcare policies and resource allocation strategies to enhance the quality and efficiency of outpatient services in rehabilitation medicine departments.

### Partial dependence plot for key features and analysis of their impact on model predictions

The target variable represents the preferred level of medical institution for patients in the following categories:

1. General hospital (excluding traditional Chinese medicine hospitals).
2. Specialist hospital (excluding traditional Chinese medicine hospitals).
3. Traditional Chinese medicine hospital.
4. Community health-service center.
5. Township health center.
6. Health-service station.
7. Village clinic/private clinic.
8. Elderly care institution/nursing home.
9. Other, please specify.

#### Correlation analysis of the first few factors in feature importance analysis

This study explored how patients choose different levels of medical institutions when facing physical discomfort. We specifically focused on the factors of importance in the characteristic analysis, such as cardiovascular and pulmonary diseases, tumors, and orthopedic diseases, as well as predictive variables, including the patient’s insurance status, medical history, and satisfaction with medical services. The results revealed a relationship between various factors and patients’ selection of medical institutions, which has important clinical and policy implications.

**Figure 3:**
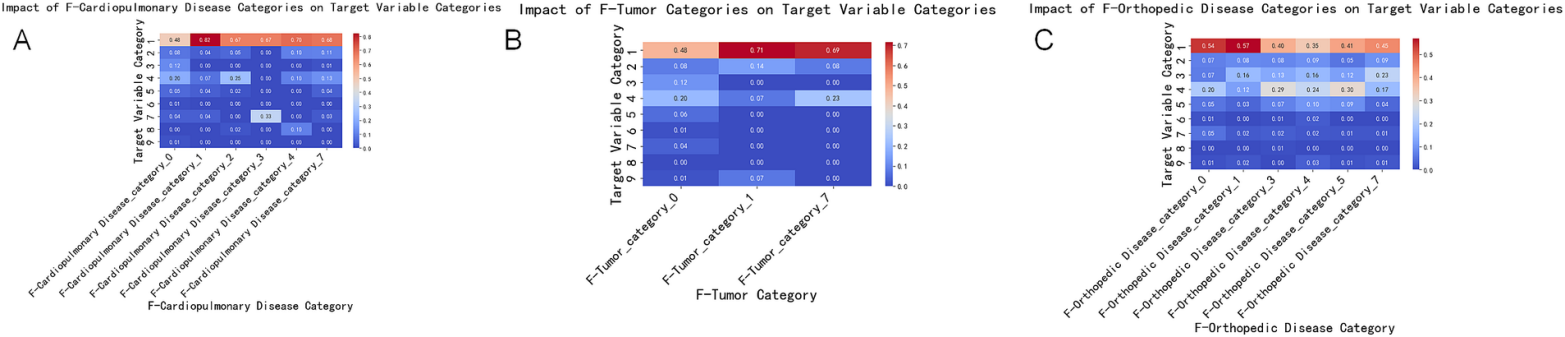
Impact of disease categories on the target variable. This set of charts shows the impact of three major disease categories (cardiopulmonary diseases, tumors, and orthopedic diseases) on the target variable categories. Each subplot depicts the extent of influence of different disease categories on the target variable, with colors ranging from blue (smaller impact) to red (greater impact). **(A)** Impact of the combined cardiopulmonary disease category (F-Cardiopulmonary Disease_combined) on the target variable categories. The X-axis represents different cardiopulmonary disease categories, and the Y-axis represents the target variable categories. The color and value of each cell indicate the extent of the disease category’s impact on the target variable categories. The meanings of different categories on the X-axis are as follows: 0 = Not having this disease, 1 = Chronic Obstructive Pulmonary Disease (COPD), 2 = Coronary Heart Disease, 3 = Myocardial Infarction, 4 = Heart Failure, 7 = Other Cardiovascular Diseases. **(B)** Impact of the combined tumor category (F-Tumor_combined) on the target variable categories. The X-axis represents different tumor categories, and the Y-axis represents the target variable categories. The color and value of each cell indicate the extent of the disease category’s impact on the target variable categories. The meanings of different categories on the X-axis are as follows: 0 = Not having this disease, 1 = Fractures, 2 = Spinal Disease, 3 = Frozen Shoulder, 4 = Lumbar Disc Herniation, 5 = Osteoarthritis, 7 = Other Orthopedic Diseases. **(C)** Impact of the combined orthopedic disease category (F-Orthopedic Disease_combined) on the target variable categories. The X-axis represents different orthopedic disease categories, and the Y-axis represents the target variable categories. The color and value of each cell indicate the extent of the disease category’s impact on the target variable categories. The meanings of different categories on the X-axis are as follows: 0 = Not having this disease, 1 = Various Tumors, 7 = Other Tumor-Related Diseases.

Relationship between diseases and the selection of comprehensive hospitals: Conditions such as cardiovascular and pulmonary diseases, various tumors, and fractures usually require high-level medical interventions, including specialized medical equipment and technology. Therefore, they are closely related to the patient’s choice to visit a comprehensive hospital. These findings emphasize the core role of comprehensive hospitals in handling complex and urgent medical needs (Figure 3).

**Figure 4:**
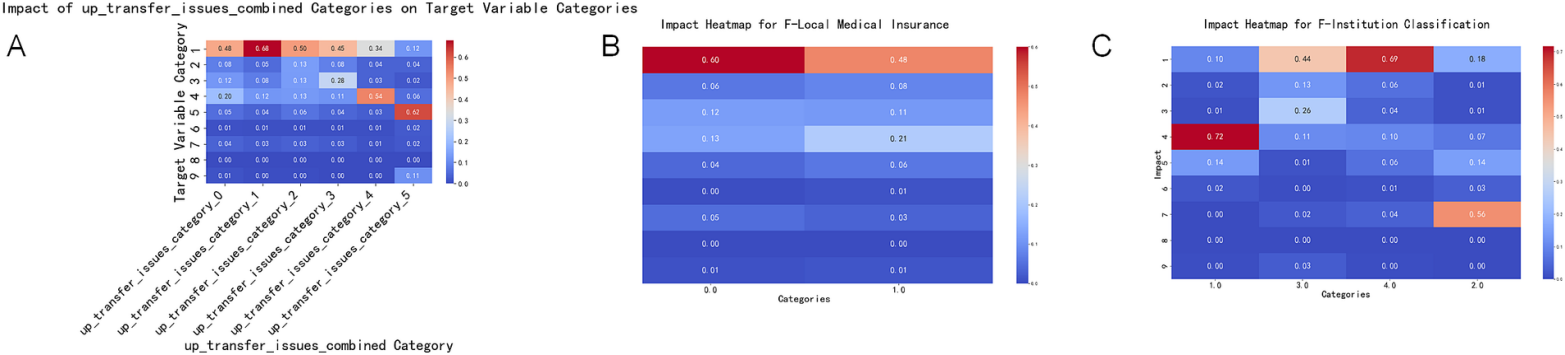
Impact of key features on the target variable. This set of charts illustrates the impact of key features (upward referral issues, local medical insurance, and institution classification) on the target variable categories. Each subplot shows the extent of influence of different feature categories on the target variable, with colors ranging from blue (smaller impact) to red (greater impact). **(A)** Impact of the combined upward referral issues category (up_transfer_issues_combined) on the target variable categories. The X-axis represents different upward referral issue categories, and the Y-axis represents the target variable categories. The color and value of each cell indicate the extent of the category’s impact on the target variable categories. The meanings of different categories on the X-axis are as follows: 0 = No issues, 1 = Unaware of upward referral, 2 = Inconvenient transportation, 3 = Dissatisfaction with higher-level hospitals’ medical expenses and reimbursement levels, 4 = Dissatisfaction with higher-level hospitals’ service attitudes, 5 = Other, please specify _______. **(B)** Impact of local medical insurance (F-Local Medical Insurance) on the target variable categories. The X-axis represents different local medical insurance categories, and the Y-axis represents the target variable categories. The color and value of each cell indicate the extent of the category’s impact on the target variable categories. The meanings of different categories on the X-axis are as follows: 0 = No, 1 = Yes. **(C)** Impact of institution classification (F-Institution Classification) on the target variable categories. The X-axis represents different institution classification categories, and the Y-axis represents the target variable categories. The color and value of each cell indicate the extent of the category’s impact on the target variable categories. The meanings of different categories on the X-axis are as follows: 1 = Community Level, 2 = Primary Level, 3 = Secondary Level, 4 = Tertiary Level.

Lack of understanding of the upward referral process and selection of comprehensive hospitals: Patients’ lack of understanding of the upward referral process may lead them to choose comprehensive hospitals directly to obtain assured treatment and diagnosis. This indicates that increasing patient awareness of the referral system may help them utilize medical resources more reasonably. Dissatisfaction with the attitude of medical services and choice of community health centers: Dissatisfaction with the attitude of higher-level hospitals may drive patients to seek more personalized and community-oriented medical services such as community health centers. This trend emphasizes the potential of community to provide higher levels of satisfaction with medical services (Figure 4A).

Insurance status and medical choices: Patients with local medical insurance tend to choose comprehensive hospitals, which may be correlated with a wide coverage range and high reimbursement rates (Figure 4B).

Current healthcare experience and future medical choices: Patients who visit primary healthcare institutions tend to choose community health centers in the future, possibly because of their trust in and satisfaction with these grassroots healthcare facilities. Patients who visited secondary and tertiary hospitals were more likely to choose comprehensive hospitals, reflecting the continued demand for advanced healthcare services. Patients who visited primary hospitals tended to choose township health clinics, possibly because of the need for basic healthcare services and geographical convenience (Figure 4C).

#### Distance, arrival time, and length of hospital stay

**Figure 5:**
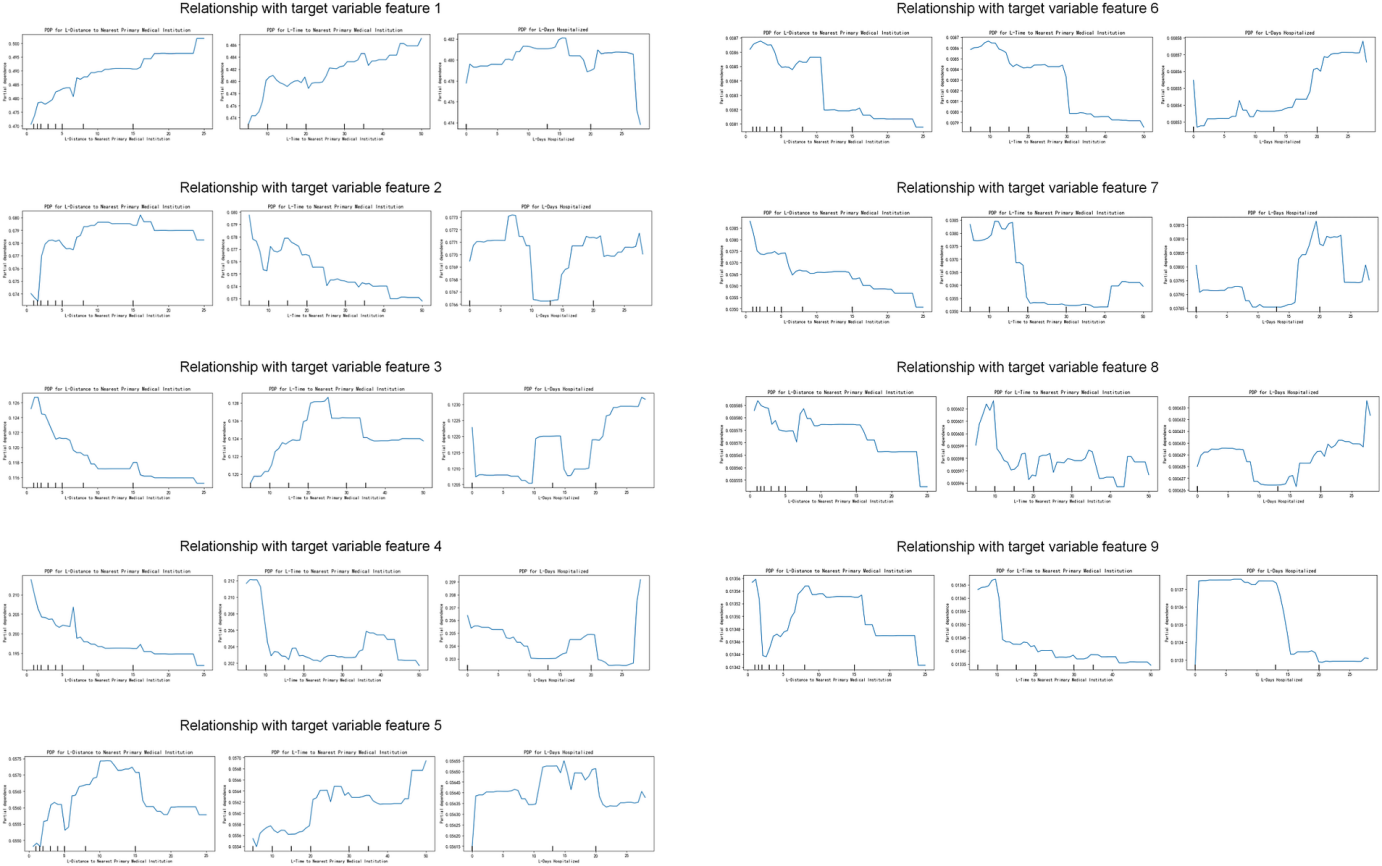
Impact of key features on the target variable. The figure shows the relationship between different features (distance to the nearest major medical institution, time to reach the nearest major medical institution, and length of hospital stay) and the target variable. Each subplot shows partial dependence plots (PDP) of specific features on different target variable characteristics, revealing the impact of feature value changes on the target variable. Features 1 to 9: Each row represents a target variable characteristic, and each column represents a feature (distance to the nearest major medical institution, time to reach the nearest major medical institution, and length of hospital stay). The X-axis represents the feature value, and the Y-axis represents the partial dependence of the target variable.

We thoroughly explored the behavioral patterns of patients choosing different types of medical institutions by plotting the dependencies between various predictive variables and the target variable. Our analysis focused on three key dimensions: the distance to the medical institution, arrival time, and length of hospital stay (Figure 5).

General Hospital: Patients who chose to go to a general hospital had a proportional relationship with distance and arrival time, particularly for those with a length of hospital stay of approximately 15 days, who tended to prefer general hospitals. This may reflect the appeal of the comprehensive services and advanced medical resources provided by general hospitals to patients who require long-term treatment.

Specialized Hospital: For specialized hospitals, distance is a significant determining factor; the closer the distance, the more likely patients are to choose this option. Additionally, we observed that patients with an average length of hospital stay of approximately 8 days tended to choose specialized hospitals more frequently, which may be related to the specialized treatments offered for specific diseases.

Chinese Medicine Hospital: Patients are more inclined toward choosing Chinese medicine hospitals if they spend 20–30 min reaching them, with common lengths of hospital stay ranging from 10 to 15 days. This indicates trends in the acceptance levels and treatment cycles of Chinese medicine.

Community Health Center: Once the arrival time exceeds 10 min, there is a significant decrease in patients’ willingness to visit community health centers, and an increase in distance acts as a deterrent. Conversely, a higher proportion of patients were willing to stay at community health centers for over 25 days, which may be related to the continuous care services provided by these centers.

Township Health Clinic: The distance that patients travel to reach a township health clinic is directly proportional to their willingness to choose treatment at the facility, reflecting the importance of township health clinics in remote areas. The average length of hospital stay is 10–20 days.

Health-Service Station: In contrast to the township health clinic, the distance that patients travel to reach the health-service station is inversely proportional to their willingness to choose treatment at the facility. However, a higher proportion of patients were willing to stay for more than 25 days, indicating its role in providing basic long-term medical services.

Village Clinic: Patients tend to forego choosing the village clinic if the travel time exceeds 20 min. The length of hospital stay was approximately 20 days, reflecting its practicality in treating short-term illnesses.

Elderly Care Institution/Nursing Home: A travel time exceeding 10 min significantly reduces patients’ willingness to choose an elderly care institution/nursing home. However, these patients are often willing to undergo hospitalization for more than 25 days, possibly because of the long-term care needs of these institutions.

#### Assessment score

Based on the assessment scores, patients can be categorized as “Bedridden group,” “Domestic group,” and “Community group.” “Bedridden group” patients have poor self-care abilities, with scores below 4 indicating complete dependence and scores between 4 and 9 indicating minimal self-sufficiency. “Domestic group” patients have slightly better self-care abilities, with scores below 4 indicating partial self-sufficiency and scores between 4 and 9 indicating greater self-sufficiency. “Community group” patients have strong self-care abilities, with scores below 4 indicating basic self-sufficiency and scores between 4 and 9 indicating complete self-sufficiency.

**Figure 6:**
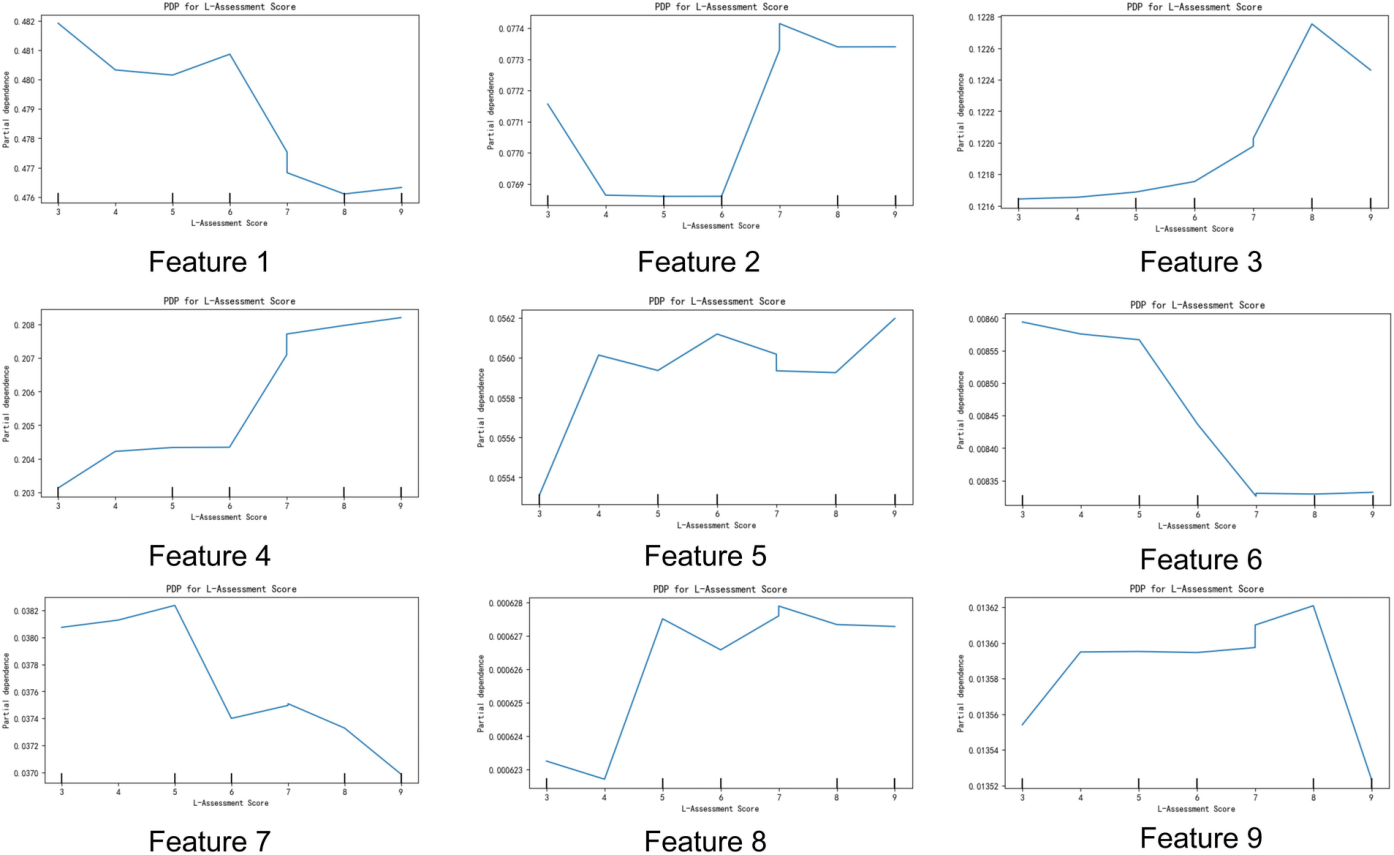
Impact of assessment scores on target variable characteristics. The figure illustrates the relationship between assessment scores (L-Assessment Score) and nine different target variable characteristics. Each subplot shows partial dependence plots (PDP) of specific target variable characteristics on the assessment scores, revealing the impact of assessment score changes on these target variable characteristics. Features 1 to 9: Each subplot represents a target variable characteristic. The X-axis represents the assessment score (L-Assessment Score), and the Y-axis represents the partial dependence of the target variable. The trend of the lines shows the impact of changes in the assessment score on specific target variable characteristics.

Regarding the choice of medical institution, the higher the assessment score, the lower the willingness to visit a general hospital. However, patients with scores above 7 were more willing to visit specialty hospitals. Patients with scores of eight preferred Chinese medicine hospitals. Higher scores indicated that patients were more inclined to choose community health centers and community health-service centers. Similarly, higher scores also made patients more willing to visit township health centers. Conversely, patients with lower scores preferred health-service stations, village clinics, and private clinics. For elderly care institutions and nursing homes, patients with higher scores showed a greater willingness to go (Figure 6).

#### Assessment outcome

In this study, we explored patients’ tendencies to choose different levels of medical institutions when facing physical discomfort, with a particular focus on the predictive variable of ‘assessment results. The assessment results reflected the daily functional self-care ability of patients, divided into six levels ranging from completely unable to self-care to fully self-reliant. Our analysis aimed to reveal the preferences of patients with different functional self-care abilities when choosing medical services, particularly their preferences for comprehensive hospitals.

Completely dependent: Patients are almost entirely unable to perform voluntary movements, with all activities confined to bed.

Largely dependent: Patients’ activities are confined to the bed, but they can perform some bed exercises voluntarily, although they cannot transfer between the bed and chair.

Partially independent: Patients can sit, stand, or transfer between bed and chair, but are limited to certain areas within their home and cannot go out independently.

Mostly independent: Patients can move freely within their home environment but cannot go out independently.

Basically independent: Patients can go out independently but have limited activity capabilities in outdoor environments.

Fully independent: Patients can perform daily activities independently without assistance from others.

Through an in-depth analysis, we found a significant correlation between patients’ functional self-care abilities and their tendency to choose general hospitals. This tendency was particularly pronounced in patients with more severe functional impairments.

Patients who are assessed as “largely dependent” (Level 2) show the highest tendency to choose general hospitals among all patients. This may be because these patients require complex medical interventions and comprehensive medical support that are typically available only in general hospitals. Following closely are patients who are “completely dependent” (Level 1), who also have a very high reliance on general hospitals due to their almost total dependence on medical support.

**Figure 7:**
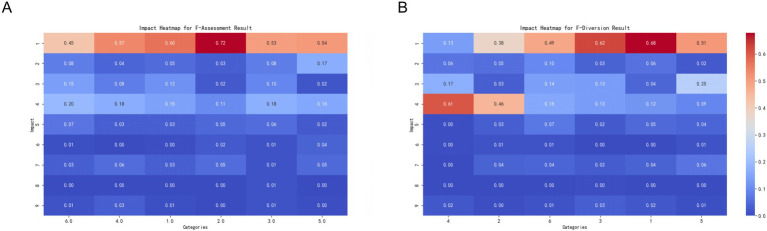
Impact of assessment and referral results on the target variable. This set of charts shows the impact of assessment results (Assessment Result) and referral results (Diversion Result) on the target variable categories. Each subplot shows the extent of influence of different categories on the target variable, with colors ranging from blue (smaller impact) to red (greater impact). **(A)** Impact of assessment results (F-Assessment Result) on the target variable categories. The X-axis represents different assessment result categories, and the Y-axis represents the target variable categories. The color and value of each cell indicate the extent of the category’s impact on the target variable categories. The meanings of different categories on the X-axis are as follows: 1 = Completely dependent (Level 1), 2 = Largely dependent (Level 2), 3 = Partially independent (Level 3), 4 = Mostly independent (Level 4), 5 = Basically independent (Level 5), 6.0 = Fully independent (Level 6). **(B)** Impact of referral results (F-Diversion Result) on the target variable categories. The X-axis represents different referral result categories, and the Y-axis represents the target variable categories. The color and value of each cell indicate the extent of the category’s impact on the target variable categories. The meanings of different categories on the X-axis are as follows: 1 = Secondary Hospitals, 2 = Nursing Homes or Elderly Care Institutions, 3 = Community Hospitals, 4 = Other Clinical Departments, 5 = Tertiary Hospitals, 6 = Outpatient Rehabilitation Treatment in Hospitals.

Patients who are “mostly independent” (Level 4) and “basically independent” (Level 5) also show a relatively high tendency to go to general hospitals, although this tendency slightly decreases as their self-care ability improves. Conversely, patients who are “fully independent” (Level 6) are the least likely to choose general hospitals, as they can usually opt for more flexible medical services, including smaller medical institutions or specialized facilities (Figure 7A).

These findings reveal not only the relationship between patients’ functional self-care abilities and their choice of medical services, but also provide important insights for medical institutions in terms of resource allocation, service optimization, and patient-oriented strategy formulation. By understanding the specific needs of patients with functional impairments, healthcare providers can respond more effectively, thereby enhancing patient satisfaction and the overall quality of healthcare services.

#### Result of diversion

This study explored how patients choose different levels of medical institutions when experiencing physical discomfort based on previous triage results. The “triage result, “serving as a predictive variable, reflects the initial medical service point to which the patient was assigned. The core aim of the analysis was to understand how these triage decisions influenced patients’ final choices of medical institutions. Triage included being directed to secondary hospitals, nursing homes or elderly care facilities, primary hospitals, other clinical departments, tertiary hospitals, and hospital rehabilitation clinics.

Relationship between patient diversion outcome and healthcare institution selection:

Other clinical departments: There was a clear tendency for patients to be diverted to other clinical departments to choose between community health and social medical centers. This may be because these patients have experienced more specialized diagnosis and treatment but still need continued support and treatment at the community level.

Primary hospitals: Patients diverted to primary hospitals also showed a strong tendency to choose community health centers or social medical centers. This suggests that although primary hospitals can provide the necessary medical services, patients may still seek further medical or health management in more convenient community environments.

Hospital rehabilitation outpatient treatment: Patients diverted to hospital rehabilitation outpatient clinics, whether from primary, secondary, or tertiary hospitals, have a strong tendency to choose comprehensive hospitals. This tendency may be due to the need for the rehabilitation of patients with comprehensive medical resources and advanced therapeutic equipment, which are usually more complete in comprehensive hospitals (Figure 7B).

These findings reveal the complex dynamics behind patient referral decisions, that is, how patients adjust their choice of healthcare institution based on their own needs and treatment effectiveness after receiving primary medical services. Particularly in rehabilitation therapy and community health services, patient decision-making reflects considerations of service quality, accessibility, and alignment with the individual health status. Understanding this phenomenon is crucial for healthcare providers and policymakers. It can help optimize resource allocation to ensure that patients receive treatment in the most suitable medical environment, as well as consider transitional needs from one service to another when designing healthcare processes.

#### Subjective judgment of doctors affects the patients’ choice of medical institutions

**Figure 8:**
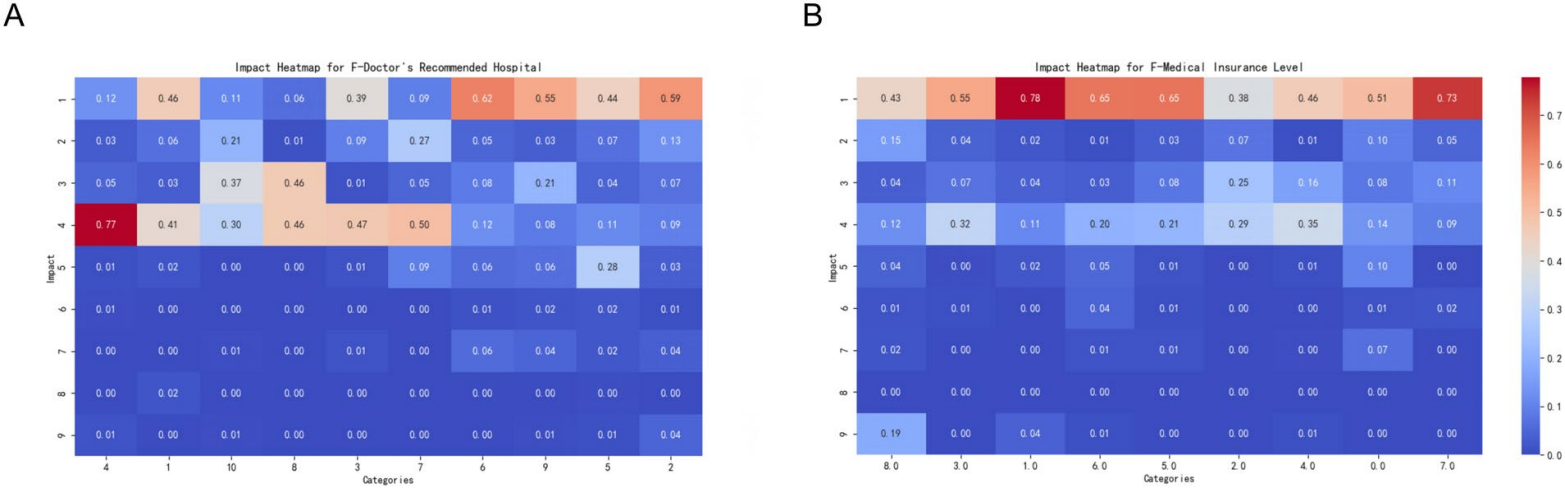
Impact of recommended hospitals and medical insurance levels on the target variable. This set of charts shows the impact of doctor’s recommended hospitals (Doctor’s Recommended Hospital) and medical insurance levels (Medical Insurance Level) on the target variable categories. Each subplot shows the extent of influence of different categories on the target variable, with colors ranging from blue (smaller impact) to red (greater impact). **(A)** Impact of doctor’s recommended hospitals (F-Doctor’s Recommended Hospital) on the target variable categories. The X-axis represents different recommended hospital categories, and the Y-axis represents the target variable categories. The color and value of each cell indicate the extent of the category’s impact on the target variable categories. The meanings of different categories on the X-axis are as follows: 1 = Secondary Medical Institutions, 2 = Inpatient at Secondary Medical Institutions, 3 = Nursing Homes or Elderly Care Institutions, 4 = Community Medical Institutions, 5 = Inpatient at Community Medical Institutions, 6 = Outpatient Rehabilitation Treatment, 7 = Other Clinical Departments, 8 = Tertiary Medical Institutions, 9 = Inpatient at Tertiary Medical Institutions, 10 = Outpatient Rehabilitation Treatment in Hospitals. **(B)** Impact of medical insurance levels (F-Medical Insurance Level) on the target variable categories. The X-axis represents different medical insurance levels, and the Y-axis represents the target variable categories. The color and value of each cell indicate the extent of the category’s impact on the target variable categories. The meanings of different categories on the X-axis are as follows: 0 = My medical insurance does not have tiers, 1 = Child Tier 2, 2 = Tier 1 Medical (employed), 3 = Tier 1 Medical (retired), 4 = Tier 2 Medical (employed), 5 = Tier 2 Medical (retired), 6 = Tier 3 Medical, 7 = Non-local flexible employment Tier 1 Medical (employed), 8 = Other, please specify.

We explored the impact of doctors’ subjective judgments on patients’ choices regarding medical institutions. By analyzing the types of medical institutions recommended by doctors to patients, we found that the actual choices made by patients are often related to doctors’ advice, but there is also a certain degree of deviation. These findings highlight the complex relationship between doctor’s recommendations and patient choices, which is of great significance for healthcare service providers and policy makers in optimizing patient referrals and guiding medical institution selection. Hospital rehabilitation outpatient department: Patients who were recommended to receive treatment at hospital rehabilitation outpatient departments tend to prefer general hospitals. Tertiary medical institutions: When doctors recommend patients to tertiary medical institutions, they tend to choose traditional Chinese medicine hospitals, community health centers, or social care centers. This finding indicates that patients seek a balance between advanced medical services and convenient community-based healthcare services. Nursing homes or eldercare facilities, primary healthcare institutions, and other clinical departments: Patients who doctors believe they should visit nursing homes or eldercare facilities, primary healthcare institutions, or other clinical departments are more likely to choose community health centers or social care centers. This may be because they found community services more convenient or suitable for receiving basic healthcare services or long-term care (Figure 8A).

Inpatient Services (Secondary, Primary, Tertiary Healthcare Institutions): When it comes to doctors’ recommendations of secondary healthcare institution inpatient services, primary healthcare institution inpatient services, outpatient rehabilitation treatment, and tertiary healthcare institution inpatient services, patients tend to choose comprehensive hospitals. This preference may reflect the high level of trust that patients have in comprehensive hospitals that provide advanced medical services and comprehensive treatment plans. These findings indicate that although doctor recommendations significantly influence patients’ choice of healthcare institution, the final decision is also influenced by multiple factors such as personal preferences, trust in specific treatment methods, and accessibility needs for medical services. Therefore, healthcare service providers should comprehensively consider these factors when formulating patient referral policies to ensure that patients receive appropriate treatment in suitable medical environments.

#### Impact of medical insurance coverage on patients’ choice of healthcare institution levels when facing physical discomfort

This study explored the impact of different levels of medical insurance on patients’ choices of healthcare institutions when faced with physical discomfort. The level of medical insurance, as a predictive variable, not only reflects the patient’s socioeconomic status and extent of insurance coverage but may also influence their acceptance and choice of healthcare services. We analyzed various levels of medical insurance, ranging from undifferentiated to multiple tiers, including different levels of medical insurance for both working and retired individuals, as well as specific medical insurance situations for non-local residents.

The relationship between the level of medical insurance and the selection of comprehensive hospitals revealed that patients at all levels showed a strong tendency to choose comprehensive hospitals. Despite potential differences in coverage and reimbursement rates for each tier, this universal inclination suggests that most insured individuals believe that comprehensive hospitals can provide more comprehensive and high-quality healthcare services, particularly when facing severe health issues (Figure 8B).

## Discussion

This study aimed to evaluate the performance of four machine learning models, XGBoost, random forest, logistic regression, and linear support vector machine (SVM), in classification tasks. The dataset used for this evaluation contained 849 samples, covering nine categories. The results indicated that ensemble learning methods outperformed single models when dealing with complex datasets. XGBoost demonstrated the best overall performance with an accuracy of 80.21%, achieving a precision of 83% and a recall of 91% in Category 1. However, its performance in Category 6 was poor, with a recall of only 9% and a precision of 33%. This may be attributed to class imbalances (Hou et al., 2020). The random forest model achieved an overall accuracy rate of 80.33%, comparable to that of XGBoost, and attained a recall rate as high as 92% for Category 1, but failed to make effective predictions for Category 8. Although random forest performs well with high-dimensional data, it may encounter challenges in handling rare categories, which could be due to insufficient node splits in the decision trees (Becker et al., 2023).

By contrast, the logistic regression model exhibited a significantly lower accuracy of only 21.67%. Its classification capabilities are poor across all categories, possibly because of the assumption of linear separability, whereas real-world datasets often contain complex nonlinear relationships; thus, it performs poorly on high-dimensional and nonlinear data (Schober and Vetter, 2021).

ROC curves were drawn to further evaluate the model performance. The AUC values of the XGBoost, random forest, and logistic regression models were 0.9147, 0.9358, and 0.6335, respectively. The higher the AUC value, the stronger the ability of the model to distinguish between positive and negative samples (Vickers and Holland, 2021). Therefore, it is evident that XGBoost and random forest exhibit significantly better classification abilities than logistic regression on this dataset. XGBoost and random forest demonstrate notable advantages in dealing with multiclass and imbalanced datasets. This study suggests that the overall performance of the XGBoost model is optimal. However, further improvements are required when handling small-scale categories. Future research should focus on enhancing the performance through data augmentation and model optimization strategies.

In this study, we conducted an in-depth investigation of the XGBoost model for multiclass classification problems, and evaluated its performance using a confusion matrix. The results demonstrate that the XGBoost model exhibits significant advantages for complex multiclass classification tasks. Specifically, the model showed outstanding classification accuracy for Categories 1 and 4, successfully identifying 391 and 116 samples, respectively. This indicated the strong identification capability of the model for important categories (Fu et al., 2022). Despite a certain degree of misclassification in other categories, the overall performance of the model demonstrated good generalization ability and robustness. The weighted voting mechanism of XGBoost and the combination of complex decision trees provided strong robustness when dealing with imbalanced datasets, as confirmed by the results of our study. Such robustness is particularly crucial for practical applications involving complex multiclass classification problems (Yang and Zhang, 2022). In this study, we selected three models—XGBoost, Random Forest, and Logistic Regression—based on their theoretical advantages, practical applicability, and suitability for our research data. XGBoost, a gradient boosting-based ensemble learning algorithm, offers efficient computational performance and powerful feature capture capabilities, particularly suitable for handling high-dimensional data and complex non-linear relationships (Dianati-Nasab et al., 2023). Random Forest combines multiple decision trees and excels in handling imbalanced data and evaluating feature importance, making it a robust non-linear classifier (Speiser et al., 2019). Logistic Regression was chosen as the baseline model due to its simplicity and interpretability, serving as a benchmark for comparing the performance and applicability of more complex models.

Furthermore, to enhance classifier performance for specific categories, especially for underperforming ones (such as Category 6), this paper also explores the potential application of more advanced classification techniques. Deep learning models, such as Convolutional Neural Networks (CNN) and Recurrent Neural Networks (RNN), have gained attention for their powerful automatic feature extraction and non-linear modeling capabilities. These models show particularly significant performance on large-scale datasets (Litjens et al., 2017). Additionally, ensemble stacking methods, by combining predictions from various base models, can effectively address class imbalance issues while improving model generalization, showing great potential for further enhancing classification performance. Future research could optimize class distribution through data augmentation (such as SMOTE) and improve model performance through hyperparameter tuning.

In this study, to address the issue of class imbalance, we utilized SMOTE (Synthetic Minority Oversampling Technique) to balance the data. SMOTE works by generating synthetic samples for the minority class to increase its representation, thereby improving the performance of the model on small classes. However, the application of this technique in the healthcare domain may affect the interpretability of the model. Since the synthetic samples generated by SMOTE are interpolations based on existing data, they may not fully reflect the actual distribution of patient characteristics in the real world. This potential bias could impact the model’s credibility in medical decision-making, especially when precise predictions for individual patients are required (Blagus and Lusa, 2013). Therefore, the use of SMOTE in healthcare applications should be approached with caution, and the validity of the generated samples should be evaluated in conjunction with domain knowledge. Furthermore, to comprehensively assess the actual effectiveness of SMOTE, we compared the performance of models with and without SMOTE. The results revealed that after applying SMOTE, the recall rate of XGBoost and Random Forest models for small classes (e.g., class 6) significantly increased (from 9 to 33%), but the precision for some larger classes slightly decreased. This suggests that while SMOTE improves class balance, it may introduce a certain degree of noise, thereby weakening the model’s predictive ability for large classes. In contrast, models without SMOTE demonstrated more stable performance on large classes but had lower recall rates for small classes, failing to meet the high sensitivity requirements for minority classes in the healthcare domain.

Based on the above analysis, we suggest that future studies using SMOTE should combine it with other techniques, such as ensemble learning or weighted loss functions, to further optimize model performance, achieving a better balance between class balance and model interpretability. Additionally, in real-world scenarios, we recommend mitigating class imbalance by expanding the dataset or incorporating more real samples, thereby reducing reliance on synthetic samples.

Through an in-depth analysis of the importance of characteristics in selecting the level of medical institutions for rehabilitation outpatients, we found that patient selection behavior is influenced by multiple factors. First, the comprehensive feature ranking of cardiovascular and pulmonary diseases was top-ranked, indicating the importance of whether patients suffer from heart- or lung-related diseases in their choice of medical institution. This is consistent with existing research demonstrating that patients with cardiovascular and pulmonary diseases generally require higher levels of medical care to address their complex health needs (Zambon and Vincent, 2008). This finding reflects the serious impact of cardiovascular and pulmonary diseases on patients’ health status, leading them to prefer medical institutions that provide specialized services.

Second, the comprehensive features of oncology and orthopedic diseases are ranked second and third, respectively, indicating that whether patients have tumors or orthopedic diseases also has a significant impact on their choice of medical institution. This may reflect the serious impact of these diseases on the patients’ physical and daily lives, making them more focused on choosing professional treatments and services (Siegel et al., 2024). For example, cancer patients may be more inclined to choose high-level medical institutions with advanced treatment equipment and technology, whereas patients with orthopedic diseases may be more concerned about the institution’s rehabilitation treatment capabilities.

In addition, the hierarchical ranking of medical institutions ranked fourth in feature importance, demonstrating the significance of patients’ awareness of the quality and expertise of medical services in influencing their selection behavior. The hierarchical system of medical institutions, through standardized evaluations of quality and service levels, affects patients’ trust in and preferences for medical institutions, thereby influencing their final choices. This finding is consistent with the trust theory in patient behavior research, indicating that an institution’s reputation and ranking significantly impact patient decision-making (Dorr Goold and Lipkin, 1999).

Through a feature importance analysis, we found that the level of medical institutions for outpatient rehabilitation medicine is influenced by multiple factors. Assessment scores also play a significant role in the selection process, in addition to individual patient health status and medical service quality. Assessment scores are based on evaluations conducted by physicians using rehabilitation grading tools to quantify a patient’s recovery needs and illness severity to help guide patients to appropriate levels in medical institutions (Stinear et al., 2020). Although assessment scores have a relatively low position in terms of feature importance as part of professional assessments by physicians, they still hold significant meaning for patients’ choices in medical institutions. This finding suggests that physicians’ professional assessments and recommendations play important roles in their decision-making processes.

These findings provide healthcare decision makers with a more comprehensive understanding, facilitating the development of more effective medical policies and resource allocation strategies. By identifying the key factors influencing patients’ choices of medical institutions, policymakers can optimize the allocation of rehabilitation medical services, thereby enhancing service quality and efficiency. For instance, specialized service capabilities and resource investments for patients with cardiovascular diseases, tumors, and orthopedic conditions could be strengthened in relevant medical institutions (Kutzin, 2013). In addition, enhancing the transparency and credibility of a tiered rating system for medical institutions will help build trust among patients regarding healthcare services.

This study identified key factors influencing patients’ choices of healthcare institutions through feature importance analysis and partial dependence plots (PDP), including facility accessibility (such as distance and travel time) and specific health conditions (such as cardiovascular diseases, cancer, and orthopedic conditions). These factors not only significantly impact patients’ healthcare decisions but may also have profound effects on their health outcomes. Firstly, facility accessibility (e.g., distance and travel time) is a crucial determinant of patients’ choices of healthcare institutions. The study found that patients are more likely to choose healthcare facilities closer to their location, especially when regular treatment or rehabilitation is required (such as community health centers or traditional Chinese medicine hospitals). However, patients requiring complex medical services (e.g., those with cardiovascular diseases or cancer) are more willing to endure longer travel times to access high-quality care at general hospitals. This pattern of choice suggests that facility accessibility not only affects patients’ access to care but may also indirectly influence their treatment adherence and eventual health outcomes. For instance, distant healthcare facilities may lead to delayed visits or treatment interruptions, negatively impacting disease management (Kelly et al., 2016). Secondly, the influence of specific health conditions on healthcare choices also holds significant clinical importance. Studies have shown that patients with cardiovascular diseases, cancer, or orthopedic conditions are more inclined to choose general hospitals or specialized hospitals, which are often equipped with advanced medical technologies and professional teams capable of delivering higher-quality diagnostic and treatment services. This preference reflects patients’ demand for high-quality care and may directly impact their health outcomes. For example, cardiovascular patients receiving timely interventions at general hospitals may experience significantly reduced rates of complications and mortality, while cancer patients receiving precise treatment at specialized hospitals may achieve better survival rates and improved quality of life (Barasa et al., 2017; Kamat et al., 2023). Additionally, other factors such as health insurance coverage and satisfaction with healthcare services also influence patients’ choices of healthcare institutions. These factors may further affect patients’ health outcomes by influencing their financial burdens and psychological experiences. Future research could explore the interactions among these factors and their impact on patients’ long-term health outcomes (Kruk et al., 2018). The findings of this study not only reveal the key factors influencing patients’ healthcare choices but also underscore their importance in clinical practice. By optimizing the allocation of healthcare resources, improving the quality of healthcare services, and enhancing patients’ trust in the healthcare system, it is possible to better meet patients’ needs and improve their health outcomes.

In the previous discussion, we explored how factors such as distance, travel time, and health conditions influence patients’ choices of healthcare institutions. These factors not only reveal patterns in patient behavior but also provide critical guidance for developing patient-centered care strategies and outpatient rehabilitation policies. Firstly, facility accessibility (e.g., distance and travel time) is a key determinant in patients’ choices of healthcare institutions. Studies have shown that patients are more likely to choose healthcare facilities that are closer or more convenient to access, especially for outpatient rehabilitation patients who require frequent visits. This finding offers practical guidance for optimizing healthcare resource allocation. For instance, in outpatient rehabilitation services, establishing community rehabilitation centers in densely populated areas or providing mobile healthcare services can reduce patients’ commuting time, thereby improving adherence to rehabilitation treatments and therapeutic outcomes. Additionally, policymakers could consider improving public transportation or offering transportation subsidies to help patients access healthcare services more easily, reducing rehabilitation interruptions or delays caused by transportation barriers (Kelly et al., 2016). Secondly, the influence of specific health conditions on patients’ healthcare choices is also of significant importance. For example, patients with chronic conditions such as cardiovascular or orthopedic diseases are more likely to seek treatment at specialized or general hospitals. This suggests that rehabilitation policies targeting these patient groups should place greater emphasis on the specialization and quality of services provided by healthcare institutions. For instance, strengthening collaboration between specialized hospitals and community rehabilitation centers, and establishing bidirectional referral mechanisms, can ensure a smooth transition for patients from acute care to community-based rehabilitation services. This approach not only enhances the efficiency of healthcare resource utilization but also meets patients’ ongoing rehabilitation needs (Kruk et al., 2018). Moreover, patient-centered care strategies should fully consider patients’ individual needs. For example, for patients with mobility limitations or those living in remote areas, telemedicine-based rehabilitation services could be promoted. By offering online consultations, rehabilitation guidance, and follow-up management, telemedicine can provide convenient, personalized care for patients. This approach not only increases patient engagement in rehabilitation but also reduces healthcare costs and alleviates disparities in the distribution of medical resources (Kruse et al., 2017). The findings of this study provide important evidence for developing patient-centered care strategies and optimizing outpatient rehabilitation policies. By improving healthcare accessibility, strengthening collaboration between healthcare institutions, and promoting telemedicine services, we can better meet patients’ needs, enhance the quality and efficiency of rehabilitation services, and drive the healthcare system toward a more patient-centered and efficient model.

In this study, we used a team-developed rehabilitation functional assessment scale to evaluate patients. This scale includes not only assessment scores but also evaluation levels to comprehensively reflect the patients’ ability to perform daily living activities. By analyzing the patients’ self-care ability assessment scores, we revealed their preferences for different medical institutions, which is of great significance in optimizing the allocation of medical resources and meeting patient needs.

The scoring tool developed in this study aims to provide a comprehensive assessment based on patients’ health conditions and key influencing factors (such as distance, travel time, and specific health conditions) to recommend the most suitable rehabilitation institution for each patient. To demonstrate the practical effectiveness of the scoring tool, we conducted a comparative analysis with existing rehabilitation assessment tools or frameworks. Existing rehabilitation assessment tools, such as the Barthel Index and the Functional Independence Measure (FIM), primarily focus on single-dimensional evaluations of patients’ functional status, such as their ability to perform activities of daily living or their level of functional independence. While these tools have played an important role in determining patients’ suitability for rehabilitation, they show limitations in helping patients choose specific rehabilitation facilities or in matching resources. Additionally, such tools often require extensive manual inputs, making them time- and labor-intensive, and less applicable in scenarios requiring rapid decision-making. In contrast, the scoring tool developed in this study integrates multiple dimensions, including patients’ functional state, individual characteristics (e.g., specific health conditions), and external environmental factors (e.g., distance and commuting time). This multidimensional approach enables quick calculation of patients’ overall scores and generates actionable recommendations for selecting an appropriate type of rehabilitation institution. This recommendation functionality is particularly valuable in real-world medical contexts, especially in resource-constrained settings or when patients need to make timely decisions. Previous studies have highlighted the significance of incorporating geographic and environmental factors, alongside patients’ health statuses, to optimize rehabilitation service decisions. For example, geographic distance and travel burdens have been shown to significantly influence patients’ adherence to rehabilitation services (Wang and Luo, 2005). Furthermore, when the scoring tool was applied to simulated test data, the results demonstrated high accuracy and validity in institution recommendations. Importantly, the tool offers detailed and interpretable outputs for personalized recommendations, such as “Patient condition requires specialized rehabilitation services at a general hospital” or “Recommended community rehabilitation services based on minimal travel burden and general rehabilitation needs.” When combined with existing assessment tools, the scoring tool not only provides insights into functional health status but also compensates for a common shortcoming in traditional frameworks—the lack of consideration for external environmental factors and accessibility (Kruse et al., 2017). By seamlessly integrating patient evaluations with institutional recommendations, the scoring tool presented in this study offers a novel, efficient, and patient-centered approach to improving access to rehabilitation services. Future research could focus on testing this tool on larger populations across diverse healthcare settings, while its integration with existing rehabilitation frameworks holds promise for refining its practicality and identifying areas for optimization.

We extensively discuss how patients choose different levels of medical institutions based on their daily functional self-care abilities when they are physically uncomfortable. As predictive variables, the assessment results differentiated six levels, from complete inability of self-care to complete self-reliance, providing crucial information for understanding patient dependency and medical needs.

An in-depth analysis showed that patients with lower self-care abilities were more inclined to choose well-equipped comprehensive hospitals, reflecting their demand for advanced medical interventions and comprehensive medical support (Porter, 2010). Particularly those who are “barely able to live independently” or “completely unable to live independently” exhibit high dependence on comprehensive hospitals because these facilities can provide necessary medical support and integrated treatment. In contrast, patients with higher self-care abilities such as those who are “mostly independent” or “partially independent,” although also inclined toward choosing comprehensive hospitals, exhibited reduced inclination as their self-reliance increased. This indicates that they may have more flexibility in selecting healthcare services and may be less reliant on advanced medical resources. Patients who are “completely independent” tend to prefer small-scale medical institutions or specialized healthcare facilities, which may be attributed to their confidence in managing daily health independently and only seeking professional healthcare assistance when specific needs arise (Bloom et al., 2018).

These findings provide valuable insights for healthcare institutions regarding resource allocation, service optimization, and the development of targeted patient-oriented strategies. Understanding patients’ self-care abilities and corresponding medical needs can help healthcare providers more accurately target services, optimize resource allocation, and improve patient satisfaction and treatment effectiveness by meeting specific needs. To further optimize medical services and resource allocation, consideration should be given to increasing the accessibility of medical facilities, particularly for patients living far from urban centers. Second, expanding and enhancing the capacity of primary healthcare services to provide continuous and comprehensive community support can alleviate the burden on large hospitals. Additionally, support should be increased for small-scale and specialized medical institutions to meet the needs of fully self-reliant patients for specialized treatment. Personalized medical planning for functionally impaired patients should be strengthened to ensure that all patients receive appropriate levels of medical care according to their specific needs. These measures will contribute to improving the overall efficiency of the tiered diagnosis and treatment.

Through analyzing the variable of “referral outcomes, “we have delved into how patients, after initially being assigned to a specific medical service point, choose subsequent medical institutions. Referral outcomes included referrals to secondary hospitals, nursing homes or elderly care facilities, primary healthcare facilities, other clinical departments, tertiary hospitals, and hospital rehabilitation clinics. These situations provide important insights into patients’ preferences for continued medical care after initial contact (Potappel et al., 2019). Research indicates that patients referred to other clinical departments tend to have a higher likelihood of choosing community health centers or community health-service centers. This may be because these patients undergo more specialized diagnosis and treatment but still require ongoing support and treatment at the community level (Kinchen et al., 2004). Furthermore, those initially referred to primary health care facilities showed a stronger inclination to seek continuous medical or health management in the community environment. This reflects the crucial role of primary healthcare services in providing a continuity of care and convenience. Patients referred to hospital rehabilitation clinics from primary healthcare facilities or secondary or tertiary hospitals demonstrated a strong tendency to choose a comprehensive hospital. This is possibly because rehabilitation patients require comprehensive medical resources and advanced treatment equipment that are usually better available at comprehensive hospitals (Potappel et al., 2019).

The efficiency of hierarchical diagnosis and treatment can be improved by optimizing the allocation of medical resources. First, it is essential to strengthen the coordination and information sharing among hospitals to ensure that patients are transferred to facilities best suited to their medical needs in a timely manner. Second, the service capabilities and facilities of primary medical institutions should be enhanced so that they can handle more nonemergency cases, thereby alleviating the burden on large hospitals. In addition, the completeness and accessibility of community medical services should be enhanced, particularly by providing more support to patients with chronic diseases who require continuous medical management. Finally, for rehabilitation services with high demand, it is recommended to establish high-quality rehabilitation facilities at multiple levels of medical institutions to ensure that patients receive the necessary rehabilitation treatment close to home. Through these measures, patient satisfaction can be improved, and the overall operational efficiency and resource utilization of the healthcare system can be effectively enhanced.

This study provides an in-depth analysis of the complex dynamics underlying patient diversion decisions, revealing how patients adjust their choice of medical institution based on their own needs and treatment outcomes after receiving initial medical services. These findings are highly valuable for medical service providers and policymakers as they can help optimize resource allocation to ensure that patients receive treatment in the most appropriate medical environment. These findings can inform the design of medical service processes by considering the need for patients to transition from one type of service to another (Dunne et al., 2023).

In addition, this study explored the impact of different health insurance coverage levels on patients’ choice of medical institutions, particularly when facing health issues and selecting hospital levels. We analyzed various health insurance tiers, from those without specific welfare benefits to those providing multiple tiers of coverage for employed, retired, and non-local residents, and found that health insurance levels significantly influenced medical choices. Research has shown that patients across all health insurance tiers tend to choose comprehensive hospitals, a preference that is closely linked to the coverage content and reimbursement rates provided by health insurance. This suggests that regardless of socioeconomic status or specific terms of health insurance, patients generally believe that comprehensive hospitals can provide higher quality and more comprehensive medical services, particularly when faced with serious health issues (Ellis and McGuire, 1993). Additionally, higher-tier health insurance categories such as those for employed individuals and retirees are typically associated with better access to healthcare resources, which may increase the likelihood of choosing comprehensive hospitals (Bloom et al., 2018).

To further optimize the medical system, we can take the following measures: First, we can enhance the flexibility and inclusiveness of medical insurance policies to ensure that necessary medical coverage is accessible to all groups, particularly low-income and uninsured populations. Second, a tiered system of medical services can be improved by enhancing the quality and capacity of primary healthcare institutions, reducing unnecessary hospital referrals and wastage of medical resources. Additionally, it encourages the establishment of more community health-service centers to provide convenient and cost-effective medical options to alleviate the burden on large hospitals. Finally, policymakers should consider using information technology to optimize patient flow systems and predict healthcare demand and resource allocation through data analysis to achieve optimal management of healthcare services (Rathnayake and Clarke, 2021). These measures will help improve overall efficiency and patient satisfaction within the medical system while ensuring that the central role of healthcare design in public health management is fully realized (Victoor et al., 2012).

The data in this study were collected from specific regions in China. Although it includes a variety of healthcare institutions and patient groups to a certain extent, there are still limitations. For instance, China’s hierarchical healthcare system, cultural background, and healthcare insurance policies may uniquely influence patients’ medical choice behaviors, restricting the generalizability of the findings to other countries or regions with different healthcare systems. Additionally, differences in the level of healthcare facilities between regions (e.g., resource-rich medical services in economically developed areas versus resource-scarce conditions in underdeveloped regions) and variations in patients’ demographic characteristics (e.g., socioeconomic status and education level) may introduce biases, potentially affecting the model’s stability and generalizability.

To address these issues, future research should broaden the scope of data sources to include patient data from more countries and regions. Regional stratified analyses should also be conducted to verify the universal applicability of the model and minimize biases introduced by region-specific characteristics (Liu et al., 2022; Say et al., 2022).

To protect patient privacy and ensure compliant data usage, this study implemented multiple measures during the data preprocessing stage. All patient data were strictly anonymized, with direct identifiers (such as names and ID numbers) and other potentially identifiable information (such as detailed addresses) removed. Additionally, encryption technology was employed during data storage and transmission to ensure data security and prevent unauthorized access. Furthermore, this study received approval from the Ethics Committee and obtained written informed consent from all participants. Patients were fully informed about the study’s purpose, the scope of data usage, and the measures taken to ensure data security. In terms of data processing, a systematic review of missing and abnormal values was conducted. Appropriate methods, such as mean imputation or deletion of incomplete records, were applied to handle these issues, minimizing data bias while ensuring the authenticity and integrity of the data. These measures highlight the study’s strong commitment to patient privacy protection and ethical compliance, while maximizing the scientific rigor and reliability of the data (Chevrier et al., 2019).

This study proposes a machine learning tool that not only enhances medical efficiency in well-equipped large healthcare institutions but is also applicable in resource-limited clinics or primary healthcare settings. In resource-constrained clinics, where there is a shortage of medical professionals, insufficient training, and a lack of adequate equipment, machine learning tools can provide decision support to assist doctors in reducing diagnostic errors and optimizing treatment plans. For example, lightweight machine learning models can enable rapid screening for common diseases using low-cost computing devices such as tablets or smartphones. Additionally, these tools can connect to cloud-based platforms, allowing small clinics to upload cases to higher-tier medical centers for further recommendations, thereby narrowing the urban–rural healthcare ga. However, implementing machine learning tools in such environments presents challenges, such as insufficient data collection, model transferability across diverse settings, the stability of network infrastructure, and healthcare professionals’ acceptance of the technology. To expand the practical impact of these tools, further optimization of the model design is needed to ensure efficient operation on low-computing-power devices and to localize outputs to accommodate varying populations or environments. Additionally, training mechanisms should be developed to improve healthcare workers’ understanding of and trust in machine learning technologies. By addressing these challenges, the tool has the potential to play a greater role in primary care and resource-limited clinics, ultimately enhancing the universality and practicality of the healthcare system (Esteva et al., 2017; Pinsky et al., 2024).

To further enhance the practicality and universality of the model, this study explores its integration with Electronic Health Record (EHR) systems and other digital health platforms. In modern healthcare systems, EHRs have become an indispensable component of clinical workflows, containing extensive structured and unstructured patient data. By integrating the machine learning model developed in this study into EHR systems, real-time data analysis and intelligent decision support can be achieved. For example, when physicians input patient symptoms or laboratory test results, the model can automatically run and provide instant diagnostic recommendations, treatment plans, and even predictions of future risks, significantly improving clinical efficiency and decision-making quality. Moreover, the integration can also enable the model to continuously refine its algorithm performance through the analysis of historical data from the EHR system, tailoring its functionality to meet the specific needs of hospitals and the unique characteristics of patients. In broader applications, the model can be embedded into digital health platforms, such as telemedicine systems or mobile health applications, to facilitate online consultations, screenings, or health monitoring services. For instance, when managing chronic diseases, the model can combine real-time dynamic metrics collected by wearable devices (e.g., blood pressure, heart rate) to provide personalized health recommendations and trigger risk alerts. However, this integration faces challenges in real-world applications, such as the heterogeneity and interoperability issues of EHR data, insufficient standardization of data formats, and considerations regarding data access permissions and privacy protection, all of which could affect the effective integration and utilization of the model. To address these challenges, future research needs to promote the implementation of data standardization technologies, strengthen the development of interfaces between EHR systems and third-party models, and ensure strict compliance with policies and regulations related to patient privacy and data security during the integration process. These optimization measures will further enhance the model’s applicability in clinical settings and support the long-term goals of healthcare intelligence and personalization (Bates et al., 2014; Jiang et al., 2017).

## Conclusion

This study evaluated XGBoost, random forest, logistic regression, and a linear SVM for classification tasks. Ensemble methods outperformed single models but struggled with class imbalance, whereas logistic regression showed lower accuracy owing to limitations with nonlinear data. Future research should expand datasets, explore hybrid models, enhance feature engineering, and conduct real-world tests. The analysis also revealed that disease type and healthcare service level significantly impacted patient preferences, particularly for cardiovascular, pulmonary, oncological, and orthopedic conditions. Tiered diagnostic and treatment tools help doctors assess patient conditions and choose the most suitable medical institutions. These insights can inform policies for optimizing resource allocation, improving service quality, and enhancing patient satisfaction and system efficiency.

## Data Availability

The datasets analyzed for the current study are not publicly available due to ethical restrictions related to the consent given by participants at the time of study commencement. An ethically compliant dataset may be made available by the corresponding author upon reasonable request and with approval from the Second People’s Hospital of Shenzhen Ethical Review Board. Requests to access the datasets should be directed to ylwang668@163.com.

## Footnotes

^1^ https://pandas.pydata.org/

^2^ https://scikit-learn.org/stable/index.html

^3^ https://imbalanced-learn.org/stable/

## Supplementary material

Supplementary Figure 1 Scores of Cross-Validation Folds. The blue bars represent accuracy, and the pink bars represent the macro F1 scores. The blue dashed line and red dashed line indicate the overall average accuracy (0.7877) and the average F1 score (0.6284), respectively.
