# Supplementary figures and images for: Prediction of outpatient rehabilitation patient preferences and optimization of graded diagnosis and treatment based on XGBoost machine learning algorithm
Source: Front Artif Intell. 2025 Jan 15;7:1473837. doi: 10.3389/frai.2024.1473837 (PMC11776094; doi:10.3389/frai.2024.1473837)

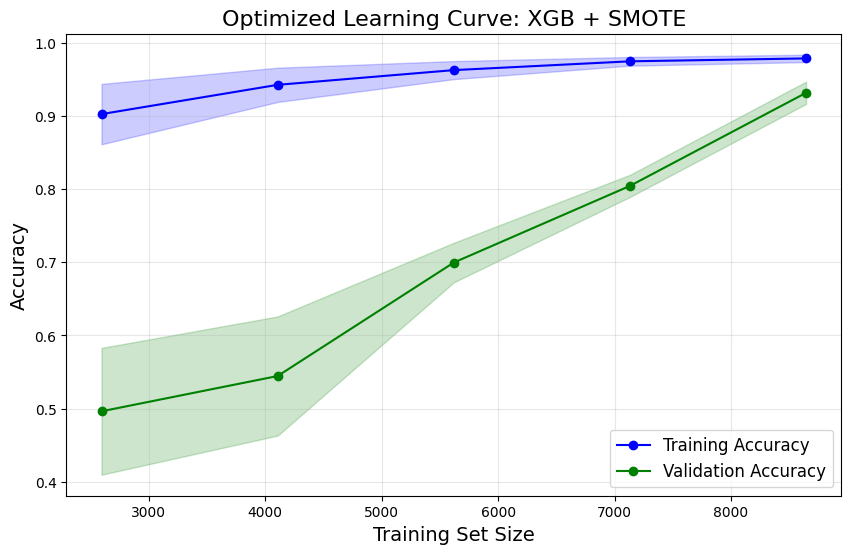

Supplement: Supplementary Figure 1 — Scores of Cross-Validation Folds. The blue bars represent accuracy, and the pink bars represent the macro F1 scores. The blue dashed line and red dashed line indicate the overall average accuracy (0.7877) and the average F1 score (0.6284), respectively. [file Image_1.tif]

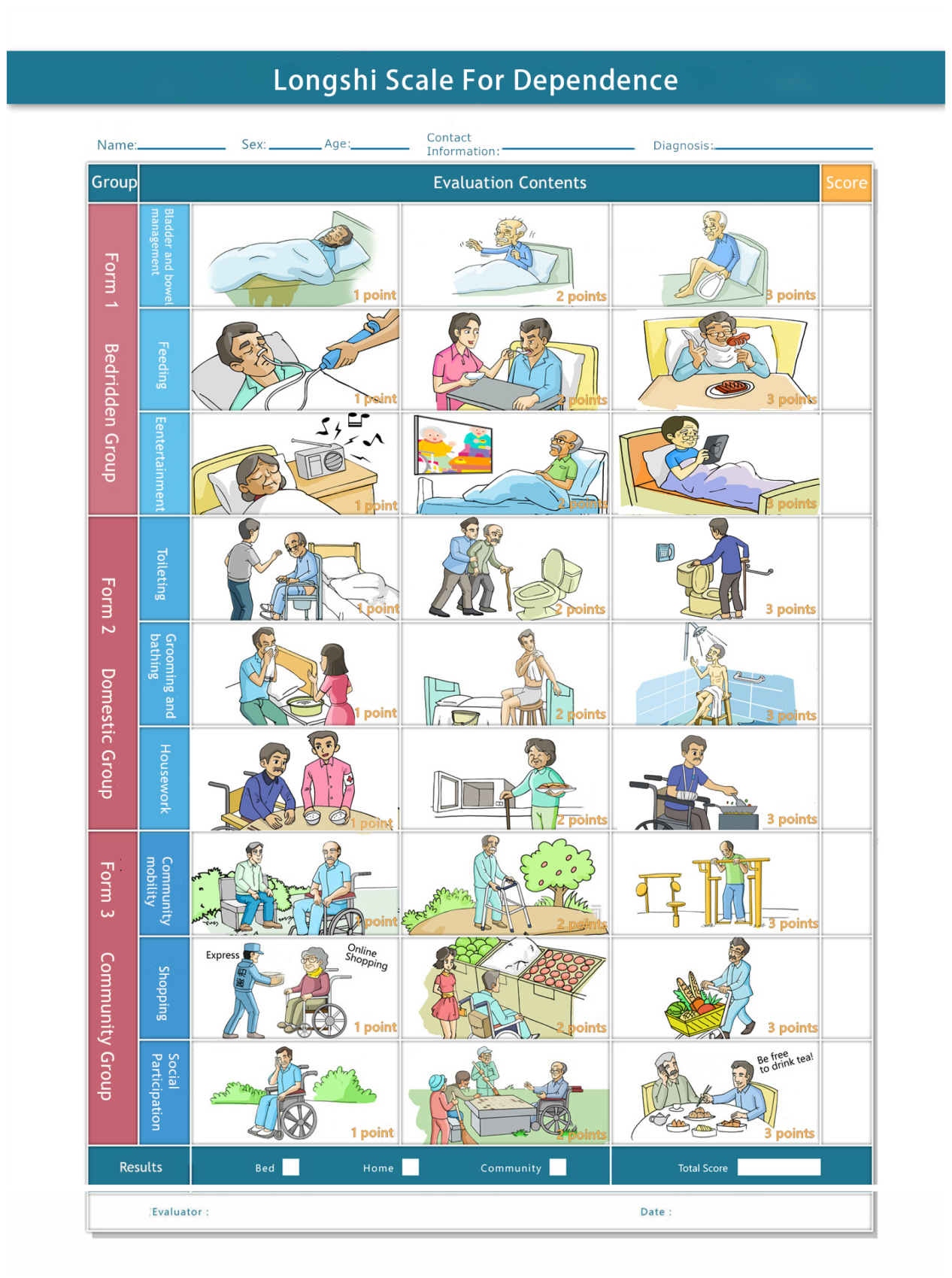

Supplement: Supplementary file 6 [file Data_Sheet_5.docx]
