# Supplementary material for: Prediction of outpatient rehabilitation patient preferences and optimization of graded diagnosis and treatment based on XGBoost machine learning algorithm
Source: Front Artif Intell. 2025 Jan 15;7:1473837. doi: 10.3389/frai.2024.1473837 (PMC11776094; doi:10.3389/frai.2024.1473837)
Supplement: Supplementary file 2 [file Data_Sheet_1.docx]

**The clinical tool for distributing rehabilitation patients**

| **Name： Sex： Age： Diagnosis：** | |
| --- | --- |
| **Indicators** | **Rehabilitation ways** |
| Patients without dysfunction | - **Non rehabilitation patients** |
| Patients with dysfunction | - **Outpatient rehabilitation treatment** |
| Community group |  |
| Stable vital signs |  |
| The disease has been controlled |  |
| Only movement disorder | - **Inpatient rehabilitation treatment:**   Primary healthcare |
| Bedridden or domestic group |  |
| Stable vital signs |  |
| The disease has been controlled |  |
| The course of disease less than 12 months |  |
| Multiple dysfunctions | - **Inpatient rehabilitation treatment:**   Secondary hospitals |
| Bedridden or domestic group |  |
| Stable or unstable vital signs |  |
| The disease has been controlled |  |
| The course of disease less than 12 months |  |
| Patients with dysfunction | - **Inpatient rehabilitation treatment:**   Tertiary hospitals |
| Bedridden or domestic group |  |
| Stable or unstable vital signs |  |
| The disease is not controlled |  |
| The course of disease less than 12 months |  |
| The functional status of patients did not change for more than one month | - **Inpatient rehabilitation treatment:**   Recommended to be referred |
| **Evaluator： Evaluate date: Copyright all rights reserved** | |
