# Supplementary material for: Prediction of outpatient rehabilitation patient preferences and optimization of graded diagnosis and treatment based on XGBoost machine learning algorithm
Source: Front Artif Intell. 2025 Jan 15;7:1473837. doi: 10.3389/frai.2024.1473837 (PMC11776094; doi:10.3389/frai.2024.1473837)
Supplement: Supplementary file 3 [file Data_Sheet_2.docx]

**Rehabilitation Graded Diagnosis and Treatment Project - Triage Assessment Questionnaire**

| **Questions** | **Answers** | | |
| --- | --- | --- | --- |
| City | 1 = Shenzhen; 2 = Mile; 3 = Hangzhou; 4 = Changzhou; 5 = Haikou; 6 = Chengdu; 7 = Tongren | | |
| Institution Name | **【Shenzhen】**  - 0 = Meilin First Village Community Health Service Center  - 1 = Sanxi Gaoyuan Community Health Service Center  - 2 = Wangmu Community Health Service Center  - 3 = Xinda Community Health Service Center  - 4 = Nanao People's Hospital  - 5 = Pingle Orthopedic Hospital  - 6 = Futian District Second People's Hospital  - 7 = Guangzhou University of Chinese Medicine Shenzhen Hospita | **【Mile】**  - 1 = Mile First Hospital  - 2 = Mile Kangyi Rehabilitation Hospital  - 3 = Mile West One Health Center  - 4 = West One Town Qifei Village Committee Health Room  **【Hangzhou】**  - 1 = Hangzhou Linping Integrated Traditional Chinese and Western Medicine Hospital  **【Changzhou】**  - 2 = Changzhou Traditional Chinese Medicine Hospital  【**Haikou】**  - 3 = Haikou People's Hospital | **【Chengdu】**  - 1 = Chengdu Second People's Hospital  - 2 = Chengdu Chenghua District Traditional Chinese Medicine Hospital  - 3 = Chengdu Chengdong Rehabilitation Hospital  - 4 = Chengdu Xinhua Hospital  **【Tongren】**  - 1 = Tongren Traditional Chinese Medicine Hospita |
| Patient Name |  | | |
| Patient ID Number |  | | |
| Primary Assessment Code | [6-digit code] | | |
| Primary Assessment Phone | [11-digit phone number] | | |
| Gender: | 0 = Female  1 = Male | | |
| Age | _____ years | | |
| Patient type | 1 = outpatients (no admissions)  2 = outpatients with admissions  3 = patients readmitted after discharge  4 = Patients transferred from other departments to undergraduate departments  5 = other types, please indicate | | |
| Assessment stage | 1 = Outpatient Assessment  2 = first assessment after admission  3 = Inpatient assessment  4 = Discharge Assessment | | |
| Diagnosis (multiple choices allowed) | - Orthopedic diseases (fracture, spine disease, periarthritis of shoulder, lumbar disc herniation, osteoarthritis, other orthopedic diseases, please specify) - Neurological diseases (stroke, brain injury, spinal cord injury, peripheral nerve injury, other neurological diseases, please specify) - Cardiopulmonary diseases (chronic obstructive pulmonary disease, coronary heart disease, myocardial infarction, heart failure, other cardiopulmonary diseases, please specify) - Geriatric diseases (hypertension, diabetes, hyperlipidemia, Parkinson's, sarcopenia, dementia, other geriatric diseases, please specify) - Pediatric diseases (cerebral palsy, autism, developmental delay/intellectual disability, other pediatric diseases, please specify) - Tumors (various tumors, other please specify) | | |
| Functional Impairment | 0 = No functional impairment  1 = Has functional impairmen | | |
| If there is impairment, please select specific types of impairment (multiple choices allowed): | - Cognitive impairment (affecting judgment of people, time, place) - Speech impairment (affecting communication) - Swallowing impairment (affecting oral intake) - Cardiopulmonary impairment (affecting limb transfer) - Motor impairment (affecting transfer of trunk, upper limbs, or lower limbs) - Other impairments, please specify: ___________ | | |
| Longshi Scale Assessment Score | Score | | |
| Longshi Scale Assessment Result | Bedridden group ( Level 1–2)  Domestic group (Level 3–4)  Community group (Level 5-6) | | |
| Duration of disease | _____ months | | |
| Is the disease under control: | 0 = Not controlled  1 = Controlled | | |
| Body temperature (armpit) | ℃  【Please set logic according to attachment 1】 | | |
| systolic pressure | mmHg  【Please set logic according to attachment 1】 | | |
| diastolic pressure | mmHg  【Please set logic according to attachment 1】 | | |
| Pulse | Time/minutes  【Please set logic according to attachment 1】 | | |
| Heart rate | Time/minutes  【Please set logic according to attachment 1】 | | |
| Breathe | Time/minutes  【Please set logic according to attachment 1】 | | |
| Pulse oxygen | %  【Please set logic according to attachment 1】 | | |
| Are the patient's vital signs stable？ | 0 = Unstable 【Please programmer settings: if the above 6 indicators, any of the indicators beyond the normal range, the automatic output of“Vital signs unstable”】  1 = Stable【Please set the programmer: if the above 6 indicators are stable in the normal range, the automatic output of“Stable vital signs”】 | | |
| Diversion Result | Hospital rehabilitation outpatient  Other clinical departments  Primary medical institution  Secondary medical institution  Tertiary medical institution  Nursing home or elderly care institution | | |
| Where the doctor thinks the patient should go (Secondary Assessment) | Secondary Medical Institutions  Inpatient at Secondary Medical Institutions  Nursing Homes or Elderly Care Institutions  Community Medical Institutions  Inpatient at Community Medical Institutions  Outpatient Rehabilitation Treatment  Other Clinical Departments  Tertiary Medical Institutions  Inpatient at Tertiary Medical Institutions  Outpatient Rehabilitation Treatment in Hospitals | | |
