# Supplementary material for: Prediction of outpatient rehabilitation patient preferences and optimization of graded diagnosis and treatment based on XGBoost machine learning algorithm
Source: Front Artif Intell. 2025 Jan 15;7:1473837. doi: 10.3389/frai.2024.1473837 (PMC11776094; doi:10.3389/frai.2024.1473837)
Supplement: Supplementary file 4 [file Data_Sheet_3.docx]

Rehabilitation Graded Diagnosis and Treatment Project - Patient Questionnaire

Patient Questionnaire Code:

[Please set an 8-digit code, an error will occur if it is more or less than 8 digits]

Is the questionnaire answered by the patient?

(1 = Yes [If yes, skip to the question about the city]; 0 = No)

If not answered by the patient, why?

(1 = Child unable to answer, answered by guardian; 2 = Patient has hearing/speech/cognitive/consciousness impairment, answered by family member; 3 = Other, please specify _______)

| Section A: Demographic Characteristics of the Patient | | | | | | |
| --- | --- | --- | --- | --- | --- | --- |
|  | **Question** | **Option** | | | **Answer** | |
|  | City | 1 = Shenzhen;  2 = Mile;  3 = Hangzhou;  4 = Changzhou;  5 = Haikou  6 = Chengdu;  7 = Tongren | | |  | |
|  | Institution | **[Shenzhen]**  0 = Meilin First Village Community Health Service Center  1 = Sanxi Gaoyuan Community Health Service Center  2 = Wangmu Community Health Service Center  3 = Xinda Community Health Service Center  4 = Nanao People's Hospital  5 = Pingle Orthopedic Hospital  6 = Futian District Second People's Hospital  7 = Guangzhou University of Chinese Medicine Shenzhen Hospital  **[Mile]**  1 = Mile First Hospital  2 = Mile Kangyi Rehabilitation Hospital  3 = Mile West One Health Center  4 = West One Town Qifei Village Committee Health Room | **[Hangzhou]**  1 = Hangzhou Linping Integrated Traditional Chinese and Western Medicine Hospital  **[Changzhou]**  2 = Changzhou Traditional Chinese Medicine Hospital  **[Haikou]**  3 = Haikou People's Hospital  **[Chengdu]**  1 = Chengdu Second People's Hospital  2 = Chengdu Chenghua District Traditional Chinese Medicine Hospital  3 = Chengdu Chengdong Rehabilitation Hospital  4 = Chengdu Xinhua Hospital  **[Tongren]**  1 = Tongren Traditional Chinese Medicine Hospital  【**Linyi** 】  1 = Linyi MCH  2=LanshanMCH 3=Hedong Rehabilitation Hospital | 【**Nanjing**】  1 = Nanjing Happy Wellness Hospital Management Co. , Ltd.  【**Changsha**】  1 = Xiangya Pok Oi Rehabilitation Hospital 【 **Jinan** 】  1 = Shandong University second hospital 【**Anshan** 】  1 = Anshan Iron & Steel Group Corporation General Hospital Qianshan Hot Spring Rehabilitation Hospital 【**Xuanwei**】  1 = Xuanwei Yunfeng Hospital 【**Kunming**】  1 = Kunming St. John Kangyuan Rehabilitation Hospital | | |
|  | ID Number | [Please set an 18-digit number, an error will occur if it is more or less than 18 digits] | | |  | |
|  | Ethnicity | 1 = Han  2 = Zhuang  3 = Manchu  4 = Hui  5 = Miao  6 = Uighur  7 = Tujia  8 = Yi  9 = Mongol  10 = Tibetan  11 = Hani  12 = Dai  13 = Bai  14 = Other, please specify _______ | | |  | |
|  | Primary Residence | 1 = Urban Center  2 = Town Center  3=Urban-Rural Junction  4 = Rural-Town Junction  5 = Rural Area | | |  | |
|  | Highest Level of Education (including adult education) | 1 = Never educated  2 = Did not complete elementary school  3 = Elementary school graduate  4 = Junior high school graduate  5 = High school graduate  6 = Technical secondary school graduate (including teacher training, vocational high school)  7 = Junior college graduate  8 = Bachelor’s degree  9 = Master’s degree  10 = Doctorate | | |  | |
|  | Whether you have retired? | 0 = Not retired  1 = retired 【skip to question 9】 | | |  | |
|  | Primary Occupation  **【**Please ask the programmer to set: only answer this question if you select 0 in question 7**】** | 0 = Unemployed  1 = Manager  2 = Professional/Technical Personnel (teacher, bank clerk, driver, electrician, etc.)  3 = Office Staff  4 = Business/Service Personnel  5 = Agricultural, Forestry, Animal Husbandry, Fishery, Water Conservancy Workers  6 = Production and Transportation Equipment Operators  7 = Self-employed  8 = Military Personnel  9 = Student  10 = Other, please specify _______ | | |  | |
|  | What was your main occupation before you retired?  **【**Please ask the programmer to set: only answer this question if you select 1 in question 7**】** | 0 = Unemployed  1 = Manager  2 = Professional/Technical Personnel (teacher, bank clerk, driver, electrician, etc.)  3 = Office Staff  4 = Business/Service Personnel  5 = Agricultural, Forestry, Animal Husbandry, Fishery, Water Conservancy Workers  6 = Production and Transportation Equipment Operators  7 = Self-employed  8 = Military Personnel  9 = Student  10 = Other, please specify _______ | | |  | |
|  | Marital Status | 0 = Never married  1 = Married, living with spouse  2 = Married, but not living with spouse temporarily due to work, etc.  3 = Married, but living apart long-term (not living as a couple)  4 = Divorced  5 = Widowed | | |  | |
|  | Number of household members living together for more than 6 months (excluding yourself) | - Grandparents: ______  - Parents: ______  - Other elder relatives: ______ (e.g., uncles, aunts)  - Siblings: ______ (including biological/step/adopted siblings)  - Spouse (fill 0 if unmarried): ______  - Number of children (fill 0 if none): ______ | | |  | |
| Section B: Diagnosis and Treatment Information  Guidance:Please share your experience regarding your diagnosis and treatment. | | | | | | |
|  | Have you heard of graded diagnosis and treatment before? (i.e., seeking treatment at different levels of institutions based on the severity and urgency of illness; or visiting a hospital for serious illnesses and a community health center for minor illnesses) | 0 = No [skip to Question 21]  1 = Yes | | |  | |
|  | How well do you understand the graded diagnosis and treatment policy?？ | 1 = Not at all  2 = Not well  3 = Average  4 = Fairly well  5 = Very well | | |  | |
|  | How well do you understand the specific implementation and procedures of graded diagnosis and treatment? | 1 = Not at all  2 = Not well  3 = Average  4 = Fairly well  5 = Very well | | |  | |
|  | Do you understand bidirectional referral?(e.g., referring from primary to secondary/tertiary institutions and vice versa) | 1 = Not at all  2 = Not well  3 = Average  4 = Fairly well  5 = Very well | | |  | |
|  | Do you understand the concept of primary diagnosis at grassroots level? | 1 = Not at all  2 = Not well  3 = Average  4 = Fairly well  5 = Very well | | |  | |
|  | Do you understand the concept of medical consortiums? | 1 = Not at all  2 = Not well  3 = Average  4 = Fairly well  5 = Very well | | |  | |
|  | Do you believe upward referral can provide effective treatment? | 1 = Not at all  2 = Not really  3 = Average  4 = Mostly  5 = Completely | | |  | |
|  | Do you believe downward referral can ensure continuous and long-term treatment? | 1 = Not at all  2 = Not really  3 = Average  4 = Mostly  5 = Completely | | |  | |
|  | Do you think common and frequently occurring diseases should be treated at grassroots medical institutions first? | 1 = Not at all  2 = Not really  3 = Average  4 = Mostly  5 = Completely | | |  | |
|  | Do you believe implementing the graded diagnosis and treatment policy can save your medical expenses | 1 = Not at all  2 = Not really  3 = Average  4 = Mostly  5 = Completely | | |  | |
|  | Do you believe implementing the graded diagnosis and treatment policy can save your consultation time? | 1 = Not at all  2 = Not really  3 = Average  4 = Mostly  5 = Completely | | |  | |
|  | Have you received or seen any promotion about graded diagnosis and treatment? | 0 = Never received/seen any promotion  1 = Community promotion (e.g., posters, neighborhood committee promotion)  2 = Hospital promotion (e.g., lectures, free clinics, doctor’s advice)  3 = Broadcast and TV reports  4 = Newspaper and magazine reading  5 = Internet information dissemination (e.g., WeChat, QQ, short video apps, other internet channels)  6 = Face-to-face chats with relatives/neighbors  7 = Other, please specify _______  8 = school publicity (such as special lectures, school teachers, etc.) | | |  | |
|  | Have you heard of family doctors? | 0 = Never heard of it  1 = Heard of it, but not signed up  2 = Heard of it, already signed up | | |  | |
|  | If you feel unwell, what type of medical institution would you choose first? | 1 = General hospital (excluding traditional Chinese medicine hospitals)  2 = Specialist hospital (excluding traditional Chinese medicine hospitals)  3 = Traditional Chinese medicine hospital  4 = Community health service center  5 = Township health center  6 = Health service station  7 = Village clinic/private clinic  8 = Elderly care institution/nursing home  9 = Other, please specify _______ | | |  | |
|  | Have you experienced downward referral before (e.g., from tertiary to secondary, secondary to primary institutions)? | 0 = No  1 = Yes | | |  | |
|  | If a doctor recommends upward referral during your consultation, would you be willing? | 1 = Completely unwilling  2 = Somewhat unwilling  3 = Average  4 = Somewhat willing  5 = Completely willing | | |  | |
|  | If a doctor recommends downward referral during your consultation, | 1 = Completely unwilling  2 = Somewhat unwilling  3 = Average  4 = Somewhat willing  5 = Completely willing | | |  | |
|  | Do you support bidirectional referrals (referring severe cases to higher-level hospitals and transferring recovering patients back to grassroots hospitals)? | 1 = Strongly support  2 = Somewhat support  3 = Average  4 = Somewhat oppose  5 = Strongly oppose | | |  | |
|  | Who do you think should have the final decision on referrals? | 1 = Doctor  2 = Patient  3 = Medical insurance organization  4 = Joint decision by doctor and patient  5 = Other, please specify _______ | | |  | |
|  | What measures do you think can improve bidirectional referrals? (multiple choices) | 1 = Change the habit of seeking treatment at large hospitals for all illnesses  2 = Establish a bidirectional referral information network platform  3 = Improve the level of grassroots hospitals  4 = Government mandates bidirectional referrals  5 = Other, please specify _______  6 = increased awareness of two-way referrals  7 = improved accessibility | | |  | |
|  | What are the main issues currently affecting your willingness to accept downward referral? (multiple choices) | 0 = No issues  1 = Unaware of downward referral  2 = Distrust of grassroots doctors’ skills  3 = Dissatisfaction with grassroots hospitals’ environment and facilities  4 = Dissatisfaction with grassroots medical expenses and reimbursement levels  5 = Other, please specify _______ | | |  | |
|  | What are the main issues currently affecting your willingness to accept upward referral? (multiple choices) | 0 = No issues  1 = Unaware of upward referral  2 = Inconvenient transportation  3 = Dissatisfaction with higher-level hospitals’ medical expenses and reimbursement levels  4 = Dissatisfaction with higher-level hospitals’ service attitudes  5 = Other, please specify _______ | | |  | |
|  | What type of medical institution do you visit most often? | 1 = General hospital (excluding traditional Chinese medicine hospitals)  2 = Specialist hospital (excluding traditional Chinese medicine hospitals)  3 = Traditional Chinese medicine hospital  4 = Community health service center  5 = Township health center  6 = Health service station  7 = Village clinic/private clinic  8 = Elderly care institution/nursing home  9 = Other, please specify _______ | | |  | |
|  | How do you rate the medical treatment level of the doctors at the medical institution you visit most often? | 1 = Very poor  2 = Poor  3 = Average  4 = Good  5 = Very good | | |  | |
|  | How do you rate the overall service attitude of the medical institution you visit most often? | 1 = Very poor  2 = Poor  3 = Average  4 = Good  5 = Very good | | |  | |
|  | How do you rate the charges at the medical institution you visit most often? | 1 = Very unreasonable  2 = Unreasonable  3 = Average  4 = Reasonable  5 = Very reasonable | | |  | |
|  | How do you rate the overall process of seeking medical treatment at the medical institution you visit most often? Including registration, consultation, examination, and payment. | 1 = Very poor  2 = Poor  3 = Average  4 = Good  5 = Very good | | |  | |
|  | How do you rate the waiting time to see a doctor at the medical institution you visit most often? | 1 = Very long  2 = Long  3 = Average  4 = Short  5 = Very short | | |  | |
|  | How do you rate the overall quality of the medical institution you visit most often? | 1 = Very poor  2 = Poor  3 = Average  4 = Good  5 = Very good | | |  | |
|  | Is treatment at the medical institution you visit most often reimbursable? | 0 = No  1 = Yes | | |  | |
|  | In the past 6 months, how many times have you visited the medical institution you visit most often? | ______ times | | |  | |
|  | Have you or your family had any unpleasant medical experiences at this institution? | 0 = No [skip to Question 43]  1 = Yes | | |  | |
|  | What unpleasant experiences have you had? (multiple choices) | 1 = Poor doctor attitude  2 = Ineffective treatment  3 = Condition worsened  4 = Too many examinations  5 = Excessive charges  6 = Other, please specify _______ | | |  | |
|  | What was the main cause of your discomfort in the past two weeks? | 1 = Hypertension  2 = Diabetes  3 = Cardiovascular diseases  4 = Chronic respiratory diseases (tonsillitis, bronchitis, lung inflammation)  5 = Chronic gastrointestinal diseases  6 = Stones (non-surgical)  7 = Rheumatic arthritis  8 = Prostate diseases  9 = Fractures (non-surgical)  10 = Cervical/lumbar diseases (non-surgical)  11 = Gynecological diseases  12 = ENT diseases (non-surgical)  13 = Other, please specify _______ | | |  | |
|  | Where was the first institution you visited when you felt unwell in the past two weeks? | 1 = General hospital (excluding traditional Chinese medicine hospitals)  2 = Specialist hospital (excluding traditional Chinese medicine hospitals)  3 = Traditional Chinese medicine hospital  4 = Community health service center  5 = Township health center  6 = Health service station  7 = Village clinic/private clinic  8 = Elderly care institution/nursing home  9 = Other, please specify _______ | | |  | |
|  | Did the doctor suggest a referral at that time? | 0 = No [skip to Question 49]  1 = Yes | | |  | |
|  | Where did the doctor suggest referring you to? | 1 = General hospital (excluding traditional Chinese medicine hospitals)  2 = Specialist hospital (excluding traditional Chinese medicine hospitals)  3 = Traditional Chinese medicine hospital  4 = Community health service center  5 = Township health center  6 = Health service station  7 = Village clinic/private clinic  8 = Elderly care institution/nursing home  9 = Other, please specify _______ | | |  | |
|  | Did you go to the suggested institution for further treatment? | 0 = No  1 = Yes [skip to Question 49] | | |  | |
|  | If not, what was the main reason? | 1 = Did not feel seriously ill  2 = Distance too far  3 = Poor family finances  4 = Medication available at home  5 = Too busy, no time  6 = Other, please specify _______ | | |  | |
|  | How long does it take to reach the nearest grassroots medical institution from your home? (Not limited to walking, cycling, bus, subway, driving; any commonly used transportation method is acceptable) | Average ______ minutes | | |  | |
|  | How far is the nearest grassroots medical institution from your home? | ______ kilometers (please specify with decimals, e.g., 0.5/3.5 kilometers, etc.) | | |  | |
| Section C: Medical Insurance and Medical Expenses  Guidance: Please fill out the information regarding your personal illness and medical history. | | | | | | |
|  | Do you currently have any of the following medical insurance? (multiple choices) | 0 = No medical insurance [skip to Question 54] (Note: If no medical insurance is selected, others cannot be selected)  1 = Urban employee medical insurance  2 = Urban and rural residents medical insurance (combined urban residents and new rural cooperative medical insurance)  3 = Urban residents medical insurance  4 = New rural cooperative medical insurance (cooperative medical)  5 = Public medical care  6 = Medical assistance  7 = Commercial medical insurance: purchased by employer  8 = Commercial medical insurance: purchased personally  9 = Major illness medical insurance for urban unemployed residents  10 = Long-term care insurance  11 = Other, please specify _______ | | |  | |
|  | What is your medical insurance tier? | 0 = My medical insurance does not have tiers  1 = Child Tier 2  2 = Tier 1 Medical (employed)  3 = Tier 1 Medical (retired)  4 = Tier 2 Medical (employed)  5 = Tier 2 Medical (retired)  6 = Tier 3 Medical  7 = Non-local flexible employment Tier 1 Medical (employed)  8 = Other, please specify _______ | | |  | |
|  | Do you have local medical insurance? | 0 = No  1 = Yes | | |  | |
|  | What is the total cost of your current visit at this institution (including insurance reimbursement and out-of-pocket expenses)? | ______ yuan [Please specify up to two decimal places] | | |  | |
|  | How much of the total cost was reimbursed by medical insurance? | ______ yuan [Please specify up to two decimal places] | | |  | |
|  | What was the total out-of-pocket expense for this visit? | ______ yuan [Please specify up to two decimal places] | | |  | |
| 59. | How much is the bed charge for this visit? (if not, fill in 0) | ______ yuan | | |  |  |
| 60. | How much is the examination fee for this visit?(if not, fill in 0) | ______ yuan | | |  |  |
| 61. | How much is the cost of your laboratory test?(if not, fill in 0) | ______ yuan | | |  |  |
| 62. | How much is the cost of your treatment ?(if not, fill in 0) | ______ yuan | | |  |  |
| 63. | How much is the cost of your operation?(if not, fill in 0) | ______ yuan | | |  |  |
| 64. | How much is the nursing fee for this visit?(if not, fill in 0) | ______ yuan | | |  |  |
| 65. | How much is the cost of sanitary materials for your visit?(if not, fill in 0) | ______ yuan | | |  |  |
| 66. | How much is the Western medicine fee for your visit?(if not, fill in 0) | ______ yuan | | |  |  |
| 67. | How much is the cost of Chinese Medicine for your visit?(if not, fill in 0) | ______ yuan | | |  |  |
| 68. | How much is the general cost of your visit?(if not, fill in 0) | ______ yuan | | |  |  |
| 69. | How much is your rehabilitation fee for this visit?(if not, fill in 0) | ______ yuan | | |  |  |
| 70. | How much is other expenses for this visit?(if not, fill in 0) | ______ yuan | | |  |  |
| **71.** | How many days have you been hospitalized at this institution (if none, fill 0)? | ______ days (if 0, skip to the next section - Family Fixed Assets) | | |  | |
| **72.** | What was the total cost of hospitalization at this institution (if none, fill 0)? | ______ yuan [Please specify up to two decimal places] | | |  | |
| **73.** | What is your annual household income? (Including the total disposable income of all family members living together, including pensions) | 1 = Less than 20,000 yuan  2 = 20,000-50,000 yuan  3 = 60,000-80,000 yuan  4 = 90,000-130,000 yuan  5 = 140,000-240,000 yuan  6 = More than 240,000 yuan | | |  | |
| **74.** | Have you experienced upward referral before (e.g., from primary to secondary or tertiary, from secondary to tertiary institutions)? | 0 = No  1 = Yes | | |  | |
| **75.** | Please fill in your phone number | [Please set an 11-digit number, an error will occur if it is more or less than 11 digits] | | |  | |
| **76.** | If there have any special circumstances that need to be explained regarding the entire  questionnaire? | 1 = Yes, please specify _______  0 = No | | |  | |
| **77.** | Name of the person filling out the form(refers to the name of the questionnaire survey personnel) | ______ | | |  | |
