# Supplementary material for: Prediction of outpatient rehabilitation patient preferences and optimization of graded diagnosis and treatment based on XGBoost machine learning algorithm
Source: Front Artif Intell. 2025 Jan 15;7:1473837. doi: 10.3389/frai.2024.1473837 (PMC11776094; doi:10.3389/frai.2024.1473837)
Supplement: Supplementary file 5 [file Data_Sheet_4.docx]

**Variable Name**

| Code |
| --- |
| F-Gender |
| L-Age |
| F-Ethnicity |
| F-Residence |
| F-Education |
| F-Occupation |
| F-Marital Status |
| F-Ancestors |
| F-City |
| F-Institution |
| F-Heard About Hierarchical Diagnosis and Treatment |
| F-Received/Seen Promotion on Hierarchical Diagnosis |
| F-Heard About Family Doctor |
| F-First Choice of Medical Institution Level |
| F-Had Downward Referral Experience |
| F-Doctor Suggested Upward Referral |
| F-Doctor Suggested Downward Referral |
| F-Support Bidirectional Referral |
| F-Final Decision Maker for Referral |
| F-Measures to Improve Bidirectional Referral |
| F-Measures to Improve Bidirectional Referral 1 |
| F-Measures to Improve Bidirectional Referral 2 |
| F-Measures to Improve Bidirectional Referral 3 |
| F-Measures to Improve Bidirectional Referral 4 |
| F-Main Issues Affecting Downward Referral |
| F-Main Issues Affecting Downward Referral 1 |
| F-Main Issues Affecting Downward Referral 2 |
| F-Main Issues Affecting Downward Referral 3 |
| F-Main Issues Affecting Downward Referral 4 |
| F-Main Issues Affecting Upward Referral |
| F-Main Issues Affecting Upward Referral 1 |
| F-Main Issues Affecting Upward Referral 2 |
| F-Main Issues Affecting Upward Referral 3 |
| F-Main Issues Affecting Upward Referral 4 |
| F-Doctor's Skill Level at Most Visited Institution |
| F-Service Attitude at Most Visited Institution |
| F-Cost at Most Visited Institution |
| F-Experience at Most Visited Institution |
| F-Waiting Time at Most Visited Institution |
| F-Overall Quality at Most Visited Institution |
| F-Reimbursement at Most Visited Institution |
| L-Visits to Institution in Past 6 Months |
| F-Unpleasant Experiences at Institution |
| F-Details of Unpleasant Experiences |
| F-Details of Unpleasant Experiences 1 |
| F-Details of Unpleasant Experiences 2 |
| F-Details of Unpleasant Experiences 3 |
| F-Details of Unpleasant Experiences 4 |
| L-Time to Nearest Primary Medical Institution |
| L-Distance to Nearest Primary Medical Institution |
| F-Participated in Medical Insurance |
| F-Participated in Medical Insurance 1 |
| F-Participated in Medical Insurance 2 |
| F-Participated in Medical Insurance 3 |
| F-Medical Insurance Level |
| F-Local Medical Insurance |
| L-Days Hospitalized |
| L-Total Hospitalization Cost |
| L-Total Medical Cost |
| L-Total Insurance Payment |
| L-Total Out-of-Pocket Cost |
| L-Annual Household Income |
| F-Understanding of Hierarchical Diagnosis and Treatment |
| F-Understanding of Bidirectional Referral |
| F-Understanding of Primary Diagnosis |
| F-Understanding of Medical Alliance |
| F-Upward Referral Effectiveness |
| F-Downward Referral Continuity of Care |
| F-Primary Care for Common Diseases |
| F-Cost Savings from Hierarchical Diagnosis Policy |
| F-Time Savings from Hierarchical Diagnosis Policy |
| F-Reason for Discomfort in Last Two Weeks |
| F-Reason for Discomfort in Last Two Weeks 1 |
| F-Reason for Discomfort in Last Two Weeks 2 |
| F-Reason for Discomfort in Last Two Weeks 3 |
| F-Reason for Discomfort in Last Two Weeks 4 |
| F-Reason for Discomfort in Last Two Weeks 5 |
| F-Reason for Discomfort in Last Two Weeks 6 |
| F-First Medical Institution for Discomfort |
| F-First Medical Institution for Discomfort 1 |
| F-Doctor Suggested Referral |
| F-Doctor Suggested Referral Location |
| F-Followed Doctor's Referral Advice |
| F-Parents |
| F-Other Relatives (e.g., Uncles, Aunts) |
| F-Siblings |
| F-Spouse |
| F-Number of Children |
| F-Had Upward Referral Experience |
| F-Had Diversion |
| F-Functional Impairment |
| F-If Impairment |
| F-If Impairment 1 |
| F-If Impairment 2 |
| F-If Impairment 3 |
| F-If Impairment 4 |
| F-If Impairment 5 |
| L-Assessment Score |
| F-Assessment Result |
| F-Disease Control |
| L-Disease Duration <= 12 Months |
| F-Patient Vital Signs Stable |
| F-Diversion Result |
| F-Doctor's Recommended Hospital |
| F-Community Province |
| F-Institution Classification |
| F-Doctor Code_y |
| F-Childhood Disease 1 |
| F-Childhood Disease 2 |
| F-Cardiopulmonary Disease 1 |
| F-Cardiopulmonary Disease 2 |
| F-Cardiopulmonary Disease 3 |
| F-Neurological Disease 1 |
| F-Neurological Disease 2 |
| F-Geriatric Disease 1 |
| F-Geriatric Disease 2 |
| F-Geriatric Disease 3 |
| F-Geriatric Disease 4 |
| F-Geriatric Disease 5 |
| F-Tumor 1 |
| F-Orthopedic Disease 1 |
| F-Orthopedic Disease 2 |
| F-Orthopedic Disease 3 |
| F-Orthopedic Disease 4 |
