# Supplementary material for: Prediction of outpatient rehabilitation patient preferences and optimization of graded diagnosis and treatment based on XGBoost machine learning algorithm
Source: Front Artif Intell. 2025 Jan 15;7:1473837. doi: 10.3389/frai.2024.1473837 (PMC11776094; doi:10.3389/frai.2024.1473837)
Supplement: Supplementary file 7 [file Data_Sheet_6.docx]

Table. The accuracy and macro-averaged F1 score of the five-fold cross-validation results.

| **Fold** | **Accuracy** | **F1-Score (Macro)** |
| --- | --- | --- |
| Fold 1 | 0.778563 | 0.573995 |
| Fold 2 | 0.789164 | 0.580057 |
| Fold 3 | 0.796231 | 0.583427 |
| Fold 4 | 0.786808 | 0.725742 |
| Fold 5 | 0.787736 | 0.678601 |
| Overall | 0.7877 ± 0.0056 | 0.6284 ± 0.0622 |
