# Supplementary material for: Prediction of outpatient rehabilitation patient preferences and optimization of graded diagnosis and treatment based on XGBoost machine learning algorithm
Source: Front Artif Intell. 2025 Jan 15;7:1473837. doi: 10.3389/frai.2024.1473837 (PMC11776094; doi:10.3389/frai.2024.1473837)
Supplement: Supplementary file 8 [file Data_Sheet_7.docx]

### **Table: Performance Metrics of the Machine Learning Model for Each Class**

| **Class** | **Precision (PPV)** | **Recall (Sensitivity)** | **Specificity** | **F1-score** | **AUC** |
| --- | --- | --- | --- | --- | --- |
| 1 | 0.83 | 0.91 | 0.98 | 0.87 | 0.95 |
| 2 | 0.79 | 0.58 | 0.95 | 0.67 | 0.82 |
| 3 | 0.80 | 0.84 | 0.96 | 0.82 | 0.89 |
| 4 | 0.74 | 0.75 | 0.94 | 0.75 | 0.85 |
| 5 | 0.84 | 0.64 | 0.97 | 0.73 | 0.88 |
| 6 | 0.33 | 0.09 | 0.99 | 0.14 | 0.72 |
| 7 | 0.64 | 0.41 | 0.98 | 0.50 | 0.77 |
| 8 | 1.00 | 1.00 | 1.00 | 1.00 | 1.00 |
| 9 | 0.83 | 0.56 | 0.99 | 0.67 | 0.79 |

### **Overall Metrics:**

- ****Accuracy****: 0.80
- ****Macro Average****: Precision = 0.76, Recall = 0.64, F1-score = 0.68
- ****Weighted Average****: Precision = 0.80, Recall = 0.80, F1-score = 0.79
