# Supplementary material for: Prediction of outpatient rehabilitation patient preferences and optimization of graded diagnosis and treatment based on XGBoost machine learning algorithm
Source: Front Artif Intell. 2025 Jan 15;7:1473837. doi: 10.3389/frai.2024.1473837 (PMC11776094; doi:10.3389/frai.2024.1473837)
Supplement: Supplementary file 9 [file Data_Sheet_8.docx]

### **Table: Definitions of Key Terms**

| **Term** | **Definition** |
| --- | --- |
| ****SMOTE**** | Synthetic Minority Oversampling Technique, a technique used to balance data class distributions by generating synthetic samples for minority classes. |
| ****ROC Curve**** | Receiver Operating Characteristic Curve, a curve used to evaluate the performance of classification models. A higher AUC value indicates better model performance. |
| ****PDP**** | Partial Dependence Plot, a visualization tool used to reveal the relationship between specific features and the target variable. |
| ****AUC**** | Area Under Curve, the area under the ROC curve, used to quantify the classification capability of a model. |
| ****Confusion Matrix**** | A tool used to evaluate the performance of classification models. It compares predicted values with actual values, helping to analyze the model’s classification results. |
